# The impact of misinformation presented during jury deliberation on juror memory and decision-making

**DOI:** 10.3389/fpsyg.2024.1232228

**Published:** 2024-01-26

**Authors:** Hayley J. Cullen, Natali Dilevski, Faye T. Nitschke, Gianni Ribeiro, Shobanah Brind, Nikita Woolley

**Affiliations:** ^1^School of Psychological Sciences, University of Newcastle, Callaghan, NSW, Australia; ^2^School of Psychological Sciences, Macquarie University, Sydney, NSW, Australia; ^3^School of Psychology, University of Sydney, Camperdown, NSW, Australia; ^4^Centre of Investigative Interviewing, Griffith Criminology Institute, Griffith University, Mt Gravatt, QLD, Australia; ^5^School of Psychology, The University of Queensland, St Lucia, QLD, Australia; ^6^School of Law and Justice, University of Southern Queensland, Ipswich, QLD, Australia

**Keywords:** juries, legal decision-making, memory, misinformation, jury deliberation

## Abstract

When deliberating, jurors may introduce misinformation that may influence other jurors’ memory and decision-making. In two studies, we explored the impact of misinformation exposure during jury deliberation. Participants in both studies read a transcript of an alleged sexual assault. In Study 1 (*N* = 275), participants encountered either consistent pro-prosecution misinformation, consistent pro-defense misinformation, or contradictory misinformation (pro-prosecution and pro-defense). In Study 2 (*N* = 339), prior to encountering either pro-prosecution or pro-defense misinformation while reading a jury deliberation transcript, participants either received or did not receive a judicial instruction about misinformation exposure during deliberation. Participants in both studies completed legal decision-making variables (e.g., defendant guilt rating) before and after deliberation, and their memory was assessed for misinformation acceptance via recall and source memory tasks. In Study 1, misinformation type did not influence legal decision-making, but pro-prosecution misinformation was more likely to be misattributed as trial evidence than pro-defense or contradictory misinformation. In Study 2, pro-defense misinformation was more likely to be misattributed to the trial than pro-prosecution misinformation, and rape myths moderated this. Furthermore, exposure to pro-defense misinformation skewed legal decision-making towards the defense’s case. However, the judicial instruction about misinformation exposure did not influence memory or decision-making. Together, these findings suggest that misinformation in jury deliberations may distort memory for trial evidence and bias decision-making, highlighting the need to develop effective safeguards for reducing the impact of misinformation in trial contexts.

## 1 Introduction

Jurors have the important task of deciding whether or not a defendant is guilty of a crime. Despite the consequences of their decisions, jurors make mistakes; innocent people can be convicted of crimes they did not commit (Huff et al., 1986; Innocence Project, 2021). For jurors to decide on cases accurately and impartially, they need to correctly remember the complex, lengthy evidence presented at trial (Ruva et al., 2007; Thorley, 2016; Hirst and Stone, 2017; Ruva and Guenther, 2017). Jurors’ memory of the evidence is a predictor of the decisions made in legal trials (Costabile and Klein, 2005). However, discussing the evidence with other jurors during deliberation may taint jurors’ memories of the evidence, allowing for inaccuracy in the jury decision-making process.

The deliberation stage of the trial is considered to be a vital component of accurate jury decision-making. Legal systems appear to hold the assumption that discussion among jurors during deliberation enhances their memory of the key details relating to the case, which leads juries to collectively reach more accurate verdicts (Pritchard and Keenan, 1999, 2002; Hirst and Stone, 2017; Jay et al., 2019). However, these assumptions are not well supported by research, as deliberation does not always assist the jury as expected (Devine, 2012). Research suggests that up to 31% of juries engage in a verdict-driven deliberation style, in which they focus on reaching a verdict rather than thoroughly evaluating the evidence (Sandys and Dillehay, 1995; Devine et al., 2004). This deliberation style may reduce the likelihood that jurors uncover mistaken interpretations or recollections of the trial evidence (Devine, 2012). Other research suggests that in group decision-making contexts, individuals are less likely to share uniquely held pieces of information (Stasser and Titus, 2003). This may mean that if jurors misremember key details of a trial and report these inaccurate details during deliberation, other jurors may not correct them or notice the mistakes.

The extensive research on erroneous eyewitness testimony sheds further light on how memory errors may occur in jury deliberations, thus distorting other jurors’ memories and interpretations. Eyewitness memory research has shown that eyewitnesses’ memories can be distorted through exposure to misinformation—incorrect information that witnesses encounter after an event (see Loftus, 2005, for a review). One way in which misinformation can be introduced is during discussion with other witnesses to the same event (Wright et al., 2000; Frenda et al., 2011). Co-witness discussion can result in memory conformity, where rather than having independent recollections of the witnessed event, witnesses’ memory reports start to influence one another’s recollections (Gabbert et al., 2003; Hope and Gabbert, 2018; Ito et al., 2019). Witnesses are more likely to produce errors in their testimony if a co-witness has introduced misinformation during a discussion, compared to if witnesses are exposed to the same misinformation through other non-social sources (Gabbert et al., 2004; Paterson and Kemp, 2006a). However, more recent research highlights the potential benefits of collaborative discussion among co-witnesses through correcting one another’s errors (see Vredeveldt et al., 2016; Vredeveldt and van Koppen, 2018). Despite these more recent findings, police officers are encouraged to prevent co-witnesses discussing an event with one another wherever practically and ethically possible, given the possible deleterious effects discussion can have on memory (Paterson and Kemp, 2005).

While discussion between witnesses to crimes has been actively discouraged in the past due to well documented issues with discussion on eyewitness memory, discussion between jurors through deliberation is instead *encouraged* (Pritchard and Keenan, 2002; Heydon, 2013). These different approaches across the criminal investigation and trial stages may exist because while eyewitnesses recall experienced events, jurors are required to make decisions about second-hand information learned during the trial. However, it is possible that akin to how witnesses may misremember key details about an eyewitness event, jurors may misremember key details from a legal trial (Pritchard and Keenan, 1999) and introduce misinformation during deliberation (Thorley et al., 2020). This misremembered information may then affect decisions made about the case. Such a possibility has received very little empirical attention (Hirst and Stone, 2017).

Misinformation presented during deliberation may lead to errors in source monitoring, thus altering jurors’ recollection of trial information. The *source monitoring framework* (Johnson et al., 1993) has been used to explain the acceptance of misinformation in an eyewitness context. This framework proposes that source monitoring errors occur because people tend to encode the content of memories without any label identifying the source. As such, it is during memory retrieval that one must decide on the source of not only the original memory, but also the misinformation. Thus, misinformation might be remembered as occurring during the original event (e.g., criminal trial) because the source of the misinformation is mistakenly believed to be the original event (Zaragoza and Lane, 1994). Furthermore, if the original event and misinformation share common characteristics, there is an increased likelihood that an individual will misattribute the source of the misinformation (Johnson et al., 1993). Because jurors actively discuss the evidence from trial, it is very likely that any case-related misinformation would share characteristics with the evidence at trial. Thus, jurors might incorrectly remember misinformation items as appearing in the trial, thereby committing a source monitoring error, because the misleading details seemingly fit with the narrative presented during the trial.

Recent research has explored whether misinformation introduced by fellow jurors during jury deliberation may lead to source monitoring errors, thereby affecting juror memory and decision-making. In Thorley et al. (2020), participants viewed a video trial, after which they read a transcript of a deliberation regarding the case. For half of the participants, the deliberation only contained correct information regarding evidence within the trial. For the other half of participants, six items of misinformation favoring the prosecution’s case were introduced into the transcript of the deliberation. Participants then provided an individual verdict, and completed a source memory test to determine whether the misinformation within the deliberation was attributed to the trial. The findings showed that those who read the deliberation containing misinformation were more likely to attribute this misinformation as evidence presented during the trial than participants who never received the misinformation. Additionally, acceptance of the misinformation impacted upon decision-making; jurors who misremembered the misinformation as real trial evidence were more likely to deliver a guilty verdict.

Thorley et al.’s (2020) findings provide preliminary evidence that not only can misinformation introduced during deliberation distort jurors’ memory for trial evidence, but that this memory distortion may impact the final verdict individual jurors decide on. However, the misinformation presented to participants during deliberation in Thorley et al.’s study only focused on the prosecution’s case, causing mock-jurors to evaluate the trial evidence in favor of the prosecution (i.e., rendering them more likely to deliver a guilty verdict). It is plausible that jurors may also mention misinformation that is consistent with the defense’s case. This may be a particular issue in sexual assault trials, where the defense case often plays to inaccurate beliefs about how sexual violence is perpetrated (sometimes called rape myths; e.g., Gray and Horvath, 2018). These inaccurate beliefs are an extra-legal factor, in that jurors are not legally permitted to consider them to make decisions in sexual assault cases (Heydon, 2013). Information which aligns with these inaccurate beliefs about sexual violence, and the defense case, may be more readily accepted by jurors (Süssenbach et al., 2012). Pro-defense misinformation may shift decision making towards the defense’s case (i.e., more acquittals than guilty verdicts). Thus, it is important to explore whether different types of misinformation would have different effects on juror memory and decision-making.

The primary aim of the current research was to explore the effect of different types of misinformation—pro-prosecution versus pro-defense—presented during jury deliberation on juror memory and decision-making. We conducted two studies to address this aim. Below, we discuss the procedure and hypotheses for Study 1.

In Study 1, participants read a fictitious trial transcript depicting an alleged sexual assault. Following this, they provided a pre-deliberation verdict, and rated the perceived credibility of the complainant’s testimony. Participants then engaged in a simulated deliberation with two other jurors, who provided misinformation on key aspects of the trial. Specifically, in the *consistent pro-prosecution* condition, both jurors consistently mentioned the same misinformation that favored the prosecution’s case; in the *consistent pro-defense condition*, both jurors consistently mentioned the same misinformation that favored the defense’s case. Given that the group size during deliberation is typically larger than groups of co-witnesses discussing a witnessed event (Paterson and Kemp, 2006b), it is also plausible that different jurors may mention misinformation for the same key detail that is contradictory (Thorley and Dewhurst, 2007; Hirst and Stone, 2017). To account for this possibility, we also included a *contradictory* condition where both jurors mentioned a different misinformation item for the same key detail; one juror’s response was consistent with the prosecution’s case, while the other juror’s response was consistent with the defense’s case. Following the deliberation phase, participants re-completed the verdict and credibility measures, as well as a free-recall and source memory test.

We hypothesized that misinformation acceptance would be greatest in both the consistent pro-prosecution and pro-defense misinformation conditions compared to the contradictory misinformation condition. There are several reasons why consistently hearing the same misinformation might lead to an increased likelihood of misinformation acceptance than hearing contradictory information. First, greater acceptance of consistent misinformation may occur because the credibility of the misinformation is heightened when it is consistently recalled by multiple sources (Mojtahedi et al., 2018; Blank et al., 2021). Second, consistent misinformation may be more likely to be accepted than contradictory misinformation because it is remembered better. Research from memory for repeated events (e.g., repeated sexual abuse) suggests that memory is stronger for details that occur in the same way across instances (e.g., the same perpetrator) and weaker memories for details that vary across instances (e.g., different forms of abuse; MacLean et al., 2018; Dilevski et al., 2020a,b; Rubínová et al., 2020a,b; Deck and Paterson, 2021a,b). Therefore, jurors presented with consistent misinformation during deliberation would be more likely to attribute that misinformation as appearing in the trial than those presented with contradictory misinformation, because memory is stronger for the former than the latter. No differences in misinformation acceptance were expected between the consistent pro-prosecution and pro-defense conditions.

As we expected the same patterns of findings for verdicts and ratings of defendant guilt, for brevity, we just report our expectations for guilt ratings below. We hypothesized that post-deliberation guilt ratings and complainant credibility ratings would be significantly higher in the consistent pro-prosecution condition than the other two conditions. However, it was unclear whether there would be a difference in post-deliberation guilt ratings and complainant credibility ratings between the consistent pro-defense and contradictory conditions, because contradictory misinformation may decrease the perceived strength of the evidence (Mojtahedi et al., 2018; Blank et al., 2021), which may reduce guilt and credibility ratings similarly to receiving misinformation that favors the defense. Finally, we hypothesized that the relationship between misinformation condition and post-deliberation ratings of guilt and credibility would be mediated by misinformation acceptance, as per the findings of Thorley et al. (2020).

## 2 Study 1 method

### 2.1 Participants

Two-hundred and ninety-eight participants took part in the study. The data from 23 participants was excluded for the following reasons: failing at least one attention check (Cullen and Monds, 2020) (*n* = 10), experiencing technical issues (*n* = 8), not completing the study (*n* = 4), or completing the study more than once (*n* = 1). This left a valid sample of 275 participants. Based on *a priori* power calculations conducting using G*Power 3.1 (Faul et al., 2007), a sample of 267 participants was required to achieve 90% power, given the design, main planned analyses (one-way ANOVAs) and assumed effect size (moderate; Lovakov and Agadullina, 2021). Participants were recruited through undergraduate research participant pools (*n* = 231), or through the paid research recruitment system of the University of Sydney (*n* = 44). Participants were required to be Australian citizens and over 18 years of age to participate in the study, to meet the basic jury eligibility requirements across all Australian states. However, Australian states have other exclusion criteria for jury service that we did not screen for (e.g., criminal history), so it should be noted that some participants may not be jury eligible depending on the jurisdiction. We also required participants to be fluent in English in order to follow and understand all study instructions. The undergraduate and paid research participation pools were based at the same institution and both used the SONA research participation platform; thus, the participants recruited through the two SONA platforms were demographically similar (gender, jury experience, English acquisition, culture). The only difference was that student participants were younger on average than paid participants, *F*(1,273) = 7.354, *p* = 0.007. Given that the samples were equivalent in all other respects, the samples were combined in all analyses. See Table 1 for the breakdown of demographic characteristics based on recruitment strategy.

**Table 1:** Demographic characteristics of Study 1 participants based on recruitment strategy.

Demographics	Student participants (*n* = 231)	Paid participants (*n* = 44)
Mean age	21.71 (6.30)	24.80 (9.52)
Gender (%)		
Female	77.5	77.3
Male	22.1	22.7
Non-binary	0.4	-
Previous jury experience (%)		
Yes	0.9	2.3
No	99.1	97.7
English as first language (%)		
Yes	81.4	79.5
No	18.6	20.5
Cultural background (%)		
European/White	52.4	36.4
East Asian	19.9	40.9
Other	27.7	22.7

“Other” for cultural background includes cultural backgrounds with low cell counts: African, Hispanic, Middle Eastern, Mixed, Pacific Islander, South Asian, Southeast Asian. Standard deviations for mean age in parentheses.

Overall, participants had a Mean Age of 22.21 years (SD = 6.99), and most participants were female (77.5%). Participants identified as the following cultural/ethnic backgrounds: European/White (49.8%), East Asian (23.3%), Southeast Asian (6.9%), mixed (6.9%), South Asian (5.5%), other (7.6%). Few participants (*n* = 3) had previously served on a jury. For most participants (81.1%), English was their first language.

### 2.2 Design

The current study employed a one-way between-subjects design with three conditions, investigating the effects of misinformation exposure (pro-prosecution vs. pro-defense vs. contradictory) on juror memory and decision-making. We made the decision not to include a pure control group that received no misinformation for two reasons. First, we wanted to ensure that we had sufficient power to detect any effects for our key research questions given practical constraints (e.g., funding, time). Second, decades of research has highlighted that exposure to incorrect information distorts memory across a variety of settings and sources (e.g., Wright et al., 2000; Loftus, 2005). The extant literature suggests that a misinformation effect would occur in a jury setting (Thorley et al., 2020); therefore, our research questions were instead centered around the factors that enhance or reduce this misinformation effect in a jury deliberation context. Participants were randomly allocated to misinformation conditions (consistent pro-prosecution *n* = 92; consistent pro-defense *n* = 89; contradictory *n* = 94). Measurements of juror memory and decision-making are described below.

### 2.3 Materials

#### 2.3.1 Trial transcript

All participants were required to read a shortened trial transcript depicting an alcohol-involved acquaintance sexual assault. More than half of sexual assaults are alcohol involved, meaning the victim and/or perpetrator have consumed alcohol (Abbey et al., 2004; Cox, 2015). As this is an early investigation of the effects of misinformation in jury deliberation, we opted to use a common type of case that jurors might be asked to consider in a criminal trial. The transcript was modified from that used in Nitschke et al. (2021). As it is common in sexual assault trials to only hear evidence from the complainant (e.g., New South Wales Law Reform Commission, 2020), our transcript features the examination-in-chief of the alleged victim, Chloe Miller, who testifies about the events leading to the sexual assault. Specifically, Chloe testifies that she was out for drinks at a bar with some work colleagues to celebrate a colleagues’ promotion. She had been casually dating the defendant, Peter Stanton, who she had met on a dating app 4 weeks prior. Peter had sent her a message to see if she was out, and the two had agreed to meet at the bar. After Peter had arrived at the bar, Chloe had a drink spilled over her, and Peter suggested that they go back to his place down the road so Chloe could clean up. Once at Peter’s apartment, Chloe and Peter had two more drinks each, and started to kiss on the sofa. Chloe started to feel uncomfortable when Peter began moving his hand up her thigh. Peter took Chloe’s clothes off and pushed Chloe down. Chloe tried to push Peter off but was unsuccessful. Peter then penetrated Chloe with his fingers and penis. The transcript was 1,534 words in length and took participants approximately 6 min, 30 s to read. Pilot testing (*n* = 15) revealed a conviction rate of 80%, and a mean guilt rating of 5.17 (out of 7) using this trial transcript.

#### 2.3.2 E-deliberation

Participants engaged in a simulated deliberation (approximately 12 min) hosted via an online, text-only chatroom. A similar method of simulated deliberation has been used in previous research (Salerno et al., 2019). Participants were led to believe that they would discuss the case with two other participants taking part in the study. However, the two other “jurors” and their associated text responses were simulated. To simulate what occurs in real legal cases, all participants were assigned a juror number prior to beginning the e-deliberation. Participants were referred to by this juror number throughout the e-deliberation. The two other “jurors” were also referred to by a number. The e-deliberation began with the “moderator” of the chatroom (also simulated) welcoming the other jurors and outlining that the purpose of the deliberation was to answer questions relating to the case. The moderator then asked eight questions about the case that all jurors answered. These questions were asked in a fixed order. The actual participant was always the first person prompted to respond to each question. Our decision to have the actual participant respond first to the question was so that their response was given prior to being exposed to misinformation. Participants could therefore not interact with or respond to the subsequent simulated responses. For four of the questions (questions 1, 3, 5, and 7), both the simulated jurors provided correct answers. For the other four questions (questions 2, 4, 6, and 8), both the simulated jurors provided incorrect answers (i.e., provided misinformation). However, the answers they provided differed depending on the experimental condition to which participants had been assigned.

Research has indicated that certain types of information influence how rape cases are perceived (e.g., Monson et al., 2000). Additionally, rape myths are often expressed throughout jury deliberations in sexual assault cases (Leverick, 2020). Common rape myths include beliefs that intoxicated victims are somewhat responsible for their rape, that a lack of resistance provides evidence against rape, and that rape cannot occur in intimate relationships (Leverick, 2020). During deliberation, if a juror misremembers the case facts in line with irrelevant rape myths (e.g., the complainant was intoxicated, did not resist, and was in an intimate relationship with the defendant), this could discredit the prosecution’s case and add credibility to the defense’s case (Dinos et al., 2015). Alternatively, if a juror misremembered the case facts in a way that opposes these rape myths (e.g., the complainant was sober, resisted, and was not in an intimate relationship with the defendant), this could have the opposite effect of adding credibility to the prosecution’s case and decreasing credibility of the defense’s case. To this end, our different misinformation conditions capture the different types of misinformation that might arise during jury deliberations for sexual assault cases, and the unique effects these types of misinformation will have on credibility and verdict.

##### 2.3.2.1 Misinformation conditions

Table 2 presents questions and responses provided by the simulated jurors based on experimental condition, and correct details for questions where misinformation was provided. Pilot testing was conducted to generate pro-prosecution and pro-defense misinformation items that were equal in similarity to the facts in the trial, to avoid any confounds across conditions.

**Table 2:** Misinformation and correct items for each question in Study 1 based on misinformation condition.

Item	Pro-prosecution	Pro-defense	Correct
Misinformation			
Status of the relationship	Just friends	Intimate relationship	Casually dating*
Length of relationship	2 weeks	6 weeks	4 weeks*
Number of drinks consumed	1 drink	3 drinks	2 drinks*
Chloe’s reaction to touching	Left hand on thigh	Moved hand up thigh	Moved hand off thigh*
Correct			
How they met			Dating app
Plan to meet on the night			Peter texted Chloe
Where they were in apartment			On the sofa
Chloe’s reaction to taking off underwear			Pushed Peter away

^*^Indicates correct details from the trial that were not featured in any version of the deliberation. These details are provided in the table to compare to the misinformation items.

###### 2.3.2.1.1 Consistent pro-prosecution

In the consistent pro-prosecution condition, both simulated jurors provided the same misinformation item, and this included information that was favorable for the prosecution case. For example, for the question: *“How long had Peter and Chloe known each other for before the night of the alleged rape?,”* both jurors in the consistent pro-prosecution condition answered 2 weeks (as opposed to the correct answer of 4 weeks).

###### 2.3.2.1.2 Consistent pro-defense

In the consistent pro-defense condition, again both simulated jurors provided the same misinformation item, but in this case the misinformation item provided information that was favorable for and served to enhance credibility of the defense’s case. For example, for the question: *“How long had Peter and Chloe known each other for before the night of the alleged rape?,”* both jurors in the consistent pro-defense condition answered that the two had known each other for 6 weeks.

###### 2.3.2.1.3 Contradictory

In the contradictory condition, for each question where misinformation was provided, one of the jurors answered with the pro-prosecution misinformation item (e.g., *“2 weeks”*), while the other answered with the pro-defense misinformation item (e.g., *“6 weeks”*).

##### 2.3.2.2 Source credibility

To determine whether perceived source credibility played a role in misinformation acceptance, participants were also asked at the end of the study to rate how accurate they believed both the jurors they deliberated with to be, on a scale from 1 (*Not at all*) to 7 (*Completely accurate*). Analyses relating to these items are presented in the Supplementary Data Sheet S1 as they are not the main focus of the study.

### 2.4 Measures

#### 2.4.1 Verdict and guilt ratings

Participants were asked to render a verdict of guilty or not guilty for the defendant with a justification. While dichotomous ratings of guilt are reflective of real jury verdicts, these measures can be less sensitive than measures of continuous guilt (Glaser et al., 2015). Therefore, participants were also asked to rate the likelihood that the defendant was guilty on a scale from 1 (*Not at all*) to 7 (*Very likely*) (Matsuo and Itoh, 2016). Participants provided their verdict and completed the guilt rating both prior to and after deliberation (i.e., before and after misinformation exposure).

#### 2.4.2 Credibility

Participants answered four questions regarding their perception of the complainant’s honesty, believability, credibility, and accuracy, on a scale from 1 (*Not at all*) to 7 (*Completely*). These questions were adapted from previous research (Connolly et al., 2008). These questions were also completed by all participants at two time points: pre- and post-deliberation. Given that we expected ratings for all four complainant questions to be similar, we checked the internal consistency of the pre- and post-ratings. These ratings revealed high internal consistency (pre-deliberation: *Cronbach’s α* = 0.892; Post-deliberation: *Cronbach’s α* = 0.926). Therefore, these ratings were aggregated to form a single pre-deliberation and post-deliberation complainant credibility score.

#### 2.4.3 Recall memory

Following the post-deliberation verdict and credibility ratings, participants’ memory was measured using a single free recall question. The free recall question asked participants to recall the key details that they regarded as most important to remember about the case. Participants were given a three-minute time limit for the free-recall task, to facilitate focusing only on the most relevant and important details about the case (including those discussed in the deliberation). The free recall task was included to examine the extent to which the misinformation and correct items presented during the deliberation would be spontaneously reported by participants as appearing during the trial.

##### 2.4.3.1 Recall coding

Participants’ free recall reports about the key details from the trial were coded to determine whether participants spontaneously mentioned the incorrect (i.e., misinformation) and correct information they encountered during the deliberation. For the current study, only misinformation and correct items presented in the deliberation were coded. For details where misinformation was provided, participants could either accept or reject misinformation items. Therefore, participants were coded as having accepted the misinformation item (i.e., reported the inaccurate misinformation they were exposed to during their deliberation), or correctly rejecting the misinformation item (i.e., reported the correct information instead of the misinformation they were exposed to during the deliberation). For example, if a participant in the pro-prosecution misinformation condition reported that the complainant and defendant had known each other for two weeks, this would be coded as “misinformation accepted.” If that same participant had reported that the complainant and defendant had known each other for 4 weeks (i.e., the correct answer), this would be coded as “misinformation correctly rejected.” Coders could not be blind to experimental conditions as misinformation acceptance depended on misinformation condition. For items where correct information was provided, participants were coded as having accepted the correct item if they reported the correct item. For example, if participants reported that Chloe and Peter had met on a dating app, this would be coded as “correct accepted”.

Two independent scorers completed the coding. Scorer 1 (HC) coded 100% of participant responses. To check for inter-rater reliability, Scorer 2 (FN) coded 50% of participant responses (*n* = 159) in line with APA publishing standards. The Intraclass Correlation Coefficients revealed moderate (*ICC* = 0.776), good (*ICC* = 0.848), and excellent (*ICC* = 0.919) reliability for misinformation accepted, misinformation correctly rejected, and correct accepted, respectively (Koo and Li, 2016). Given the acceptable reliability, the coding from Scorer 1 was used in the analyses.

#### 2.4.4 Source memory

Following the free recall report, participants completed a source memory test. Following previous jury misinformation research (Ruva et al., 2007; Thorley et al., 2020), participants read a series of statements and they were instructed that the information in the statement may have come from different sources (trial only, only deliberation, both trial and deliberation, or neither). Participants were asked to identify the source of the information, and to rely on their own memory for the source of the information. The response options were *Trial Only, Deliberation Only, Both Trial and Deliberation, and Neither Trial nor Deliberation*. These instructions were based on those provided in Mitchell and Zaragoza (2001).

The source memory test consisted of 24 items (or statements). There were 4 types of items: 8 *misinformation* (deliberation only), 4 *correct* (both trial and deliberation), 4 *correct* (trial only), and 8 *new* items. The *misinformation* items restated the misinformation presented during the e-deliberation (correct answer: *Deliberation Only*). However, for participants in the consistent misinformation conditions (pro-prosecution and pro-defense), 4 of the 8 misinformation items were technically filler items, as those items had not been presented during the e-deliberation for these conditions. These four filler items were not included in the scoring for these conditions. The misinformation items were included to provide a measure of participants’ proclivity to “misinformation acceptance” (i.e., a critical source monitoring error) after being misled about trial details during the deliberation.

The *correct information* (both trial and deliberation) items restated the correct information presented during the trial and the deliberation (e.g., *Chloe and Peter met on a dating app*) (correct answer: *Both Trial and Deliberation*). The *correct* (trial only) items restated the information presented during the “trial only” (e.g., *As Chloe and Peter kissed, Chloe moved Peter’s hand away from her thigh*). The *new* items stated information that appeared in “neither trial nor deliberation” (e.g., *Chloe and Peter first met each other through a co-worker*). These items were based on what appeared during the trial, but they suggested alternative information about what occurred. We included the correct information from deliberation, correct (trial only), and new information items to provide a measure of whether the participants across the three conditions remembered the trial equally well (Thorley et al., 2020). Analyses relating to these items are presented in the Supplementary Data Sheet S1 as they are not the main focus of the study. Overall, the analyses revealed that performance on these items did not differ across misinformation conditions.

Participants’ responses to each item in the source memory test were scored to determine whether they had misremembered/remembered the information as appearing during the trial. Specifically, participants received one point each time they had responded to a test item with ‘Trial Only’ or ‘Trial and Deliberation’, as both responses indicate that a participant remembered that the information appeared during the trial. After scoring was complete, we summed together participants’ ‘Trial Only’ and ‘Trial and Deliberation’ scores for each item type separately. For data analysis purposes, proportion scores for each information type were calculated by dividing participants’ ‘Trial Only’ and ‘Trial and Deliberation’ scores by the number of items for that information type. For example, if a participant in the pro-prosecution condition misremembered that two out of four items of misinformation appeared during the trial, their proportion score would be 0.5 (2/4 = 0.5).

Since participants in the contradictory condition received both pro-prosecution and pro-defense misinformation items, three proportion scores were computed pertaining to performance for these items. We calculated a proportion score for pro-prosecution and pro-defense misinformation items, separately. Then, for the main analysis relating to misinformation items, we calculated an average proportion score for misinformation acceptance between the pro-prosecution and pro-defense items (i.e., [proportion pro-prosecution score + proportion pro-defense score]/2).

#### 2.4.5 Attention checks and suspicion

At different stages of the study, participants were asked three instructional attention check questions to ensure that they followed the instructions (Oppenheimer et al., 2009). Participants who answered any of these questions wrong were removed from the analyses (Cullen and Monds, 2020). Participants were also asked questions at the end of the study to determine whether they were suspicious about the aims of the study. They were asked if they noticed anything strange about the study, and if so, to report what was strange (Salerno et al., 2019). This was not used as a basis for exclusion, but instead to determine whether participants were suspicious about the simulated deliberation and whether this suspicion mattered. Analyses were conducted with and without participants who were suspicious about the deliberation, to determine whether suspicion impacted upon the study results.

### 2.5 Procedure

Participants signed up for the study advertised as “Jury decision-making.” The study took place in 2020 and 2021. Thus, due to COVID-19 social distancing requirements, the study was conducted online. Once a participant had signed up to the study, an experimenter made contact with that participant via email to arrange a day and time to complete the session. At the time of each participant’s appointment, an experimenter emailed the participant the link to the online experiment. The online session began with participants providing informed consent. They were then presented with general instructions about the study. Specifically, they were informed that the study was being conducted by researchers from two different universities, and that they would read about a criminal trial, engage in a deliberation with other participants from the other institution (to increase the realism of the study and the simulated deliberation), and answer some questions about the trial.

Following the general instructions, participants read the trial transcript about a sexual assault case. To ensure that participants attended to the trial transcript, they were given a minimum of three minutes to read the transcript and could not proceed until the time had elapsed. The minimum time limit was determined through pilot testing. After reading the trial transcript, participants completed the pre-deliberation measures of verdict, guilt rating, and complainant credibility. Therefore, this pre-deliberation decision-making occurred prior to any misinformation exposure. Participants were directed via email to log into the chat room where they would engage in the live online deliberation with other participants. Participants then engaged in the 12-min e-deliberation where they either received pro-prosecution, pro-defense, or contradictory misinformation by the simulated jurors.

After the deliberation, participants completed the post-deliberation measures of verdict, guilt rating, and complainant credibility (i.e., after misinformation exposure). Participants then completed the free recall and source memory measures, and then rated how accurate they believed the two “jurors” were during the e-deliberation. Finally, participants completed the suspicion check questions and several demographic questions. Upon completion of the study, participants were fully debriefed about the study. The majority of the study was hosted using Qualtrics survey software. However, the simulated e-deliberation was hosted on AJAX chat. It took approximately 45 min to complete the study. All aspects of the study were approved by the University of Sydney Human Research Ethics Committee (protocol number: 2019/947).

### 2.6 Transparency statement

We reported how we determined our sample size, all data exclusions (if any), all manipulations, and all measures in this study. The hypotheses, design, measures, and analysis plan were pre-registered on Open Science Framework (OSF). See here for the original registration https://osf.io/kdbma/ and here for the amended version https://osf.io/2rxye/. Any deviations from the pre-registration are reported transparently below. All experimental materials (including the e-deliberation script) and data (dataset, output, and code) are available on OSF.^1^

### 2.7 Transparent deviations

First, we collected data during periods of lockdown in Australia through the COVID-19 pandemic. This meant we had to switch from lab-based participant recruitment to transform the study to be fully online. As a result, we had to switch to participants having a juror number instead of their name in the online deliberation and we also were unable to record participants’ memory responses during the online deliberation to determine their original memory prior to misinformation exposure.

Second, we planned to run mediation analyses to determine whether: (1) misinformation acceptance mediated the relationship between misinformation condition and post-deliberation measures, and (2) perceived credibility of the other jurors mediated the relationship between misinformation condition and misinformation acceptance. We did not find direct effects of misinformation condition on post-deliberation measures or perceived credibility of other jurors. Therefore, we did not run the planned mediations.

## 3 Study 1 results

### 3.1 Overview and analysis plan

First, we reported the descriptive statistics relating to suspicion about the simulated deliberation. We then moved on to decision-making. We conducted preliminary analyses (one way ANOVAs and a chi-square test) to ensure that there were no differences in pre-deliberation measures across misinformation conditions (i.e., that there were no pre-existing differences in attitudes and beliefs about the case before the misinformation was introduced). Then, one way ANOVAs were conducted using the post-deliberation measures as dependent variables, to determine whether misinformation exposure influenced juror perceptions and decision-making. Next, we focused on memory. We conducted a series of one way ANOVAs with planned contrasts to determine whether misinformation condition influenced participants’ memory and misinformation acceptance. The free recall data violated the assumption of normality, so we also conducted robust ANOVAs using 10% trimmed means (Wilcox, 2012). Results of both approaches were the same, so we report the original ANOVA results here for ease of interpretation. Finally, we used a one way ANOVA with planned contrasts to determine whether the misinformation condition affected the perceived credibility of the jurors (see Supplementary Data Sheet S1 for these analyses).

To corroborate non-significant findings, we conducted exploratory Bayesian analyses via the Bayes Factor package (Morey and Rouder, 2018) in R (version 4.0.3; R Core Team, 2020). We implemented default priors to conduct these analyses as they make few assumptions about the data and offer a conservative test of the null hypothesis (Rouder et al., 2012). Bayes Factors quantify the evidence in favor of either the null or alternate hypotheses (Rouder et al., 2012). When reporting Bayes Factors, we use the interpretations provided by Jeffreys (1961) to indicate the strength of evidence for the null or alternate hypothesis. Bayes Factors of 1–3, 3–10, 10–30, 30–100, or > 100 reflect anecdotal, moderate, strong, very strong, and extreme evidence in favor of one hypothesis over the other, respectively.

### 3.2 Suspicion about deliberation

Participants were asked whether they noticed anything strange about the study, and to elaborate if they had, to determine whether they were suspicious about the nature of the deliberation. As seen in Table 3, 14.5% of participants believed that the deliberation chatroom was simulated and thus the other jurors in the chatroom were not real people (e.g., *“The other participants in the chat were bots”*—Participant 7). A further 24% of participants thought that the other jurors in the deliberation chatroom were either confederates (e.g., *“I do not believe the other jurors were real participants and were actually confederates”*—Participant 267) or that they were real participants, but were given different scenarios (e.g., *“it appears we were given different stories, perhaps to mimic real jurors different interpretations and memories?”*—Participant 15). Additionally, 28.4% of participants thought that the jurors in the deliberation chatroom had an incorrect recollection of the scenario (e.g., *“yes the discussion did not seem accurate and it made me question my own interpretation of the trial”*—Participant 238). Finally, about a third of participants (33.1%) were not suspicious of the simulated deliberation. A chi-square test revealed a significant association between type of suspicion and misinformation condition, *χ^2^* (*N* = 275) = 18.008, *p* = 0.021, *φ_c_* = 0.181, such that participants in the contradictory group reported believing the deliberation was simulated above expected counts.

**Table 3:** Study 1—type of suspicion about the simulated deliberation, total and across misinformation conditions.

Type of suspicion	Total *n*	Pro-prosecution	Pro-defense	Contradictory	*%*
Simulated deliberation	40	10	8	22	14.5
Confederate used/Scenarios manipulated	66	16	23	27	24.0
Jurors incorrect	78	33	24	21	28.4
No suspicion	91	33	34	24	33.1

For each of the analyses reported below, we conducted the same analyses retaining just the participants who were not suspicious about the deliberation (*N* = 91). We will report when the analyses differed after accounting for suspicious participants.

### 3.3 Decision-making

Participants delivered a verdict, rated the defendant’s guilt, and rated the complainant both before and after deliberation. Before the deliberation, 87.6% of participants delivered a verdict of “guilty,” while 12.4% of participants delivered a verdict of “not guilty.” After the deliberation, 86.9% of participants delivered a verdict of “guilty,” while 13.1% of participants delivered a verdict of “not guilty.” The descriptives for the pre- and post-deliberation measures based on misinformation condition are reported in Table 4.

**Table 4:** Study 1—pre- and post-deliberation verdict, defendant guilt rating, and complainant credibility ratings across misinformation condition.

	Misinformation condition		
	Pro-prosecution (*n* = 92)	Pro-defense (*n* = 89)	Contradictory (*n* = 94)
Pre-deliberation			
% Guilt	89.1	82	91.5
Defendant guilt rating	5.57 (1.32)	5.26 (1.34)	5.59 (1.18)
Complainant credibility	5.49 (0.99)	5.33 (0.92)	5.35 (1.00)
Post-deliberation			
% Guilt	85.9	83.1	91.5
Defendant guilt rating	5.66 (1.48)	5.46 (1.42)	5.63 (1.12)
Complainant credibility	5.47 (1.09)	5.21 (1.12)	5.31 (1.10)
Pre-post deliberation			
Defendant guilt rating	−0.09 (0.81)	−0.20 (0.98)	−0.04 (0.70)
Complainant credibility	0.02 (0.48)	0.12 (0.63)	0.04 (0.57)

Values for defendant guilt rating and complainant credibility represent mean rating (standard deviation in parentheses).

At pre-deliberation, a chi-square analysis revealed no significant relation between misinformation condition and verdict, *χ^2^* (*N* = 275) = 4.066, *p* = 0.131, *φ_c_* = 0.122, BF₀₁ = 5.323. The ANOVAs revealed no significant differences in pre-deliberation guilt ratings (*F*(2,272) = 1.855, *p* = 0.158, *η_p_^2^* = 0.013, BF₀₁ = 4.834) and complainant credibility ratings (*F*(2,272) = 0.786, *p* = 0.457, *η_p_^2^* = 0.006, BF₀₁ = 12.636) based on misinformation condition. Overall, these analyses suggest that the randomization to misinformation condition was effective.

At post-deliberation, a chi-square analysis revealed no significant relation between misinformation condition and verdict, *χ^2^* (*N* = 275) = 2.928, *p* = 0.231, *φ_c_* = 0.103, BF₀₁ = 3.149. Additionally, one-way ANOVAs were conducted to explore whether differences in guilt and credibility ratings from pre- to post-deliberation differed as a function of misinformation condition. For both guilt and complainant credibility difference scores, the effect of misinformation condition was not significant (guilt: *F*(2,272) = 0.855, *p* = 0.426, *η_p_^2^* = 0.006, BF₀₁ = 11.879; credibility: *F*(2,272) = 0.842, *p* = 0.432, *η_p_^2^* = 0.006, BF₀₁ = 12.029).

### 3.4 Memory

#### 3.4.1 Free recall

**Figure 1:**
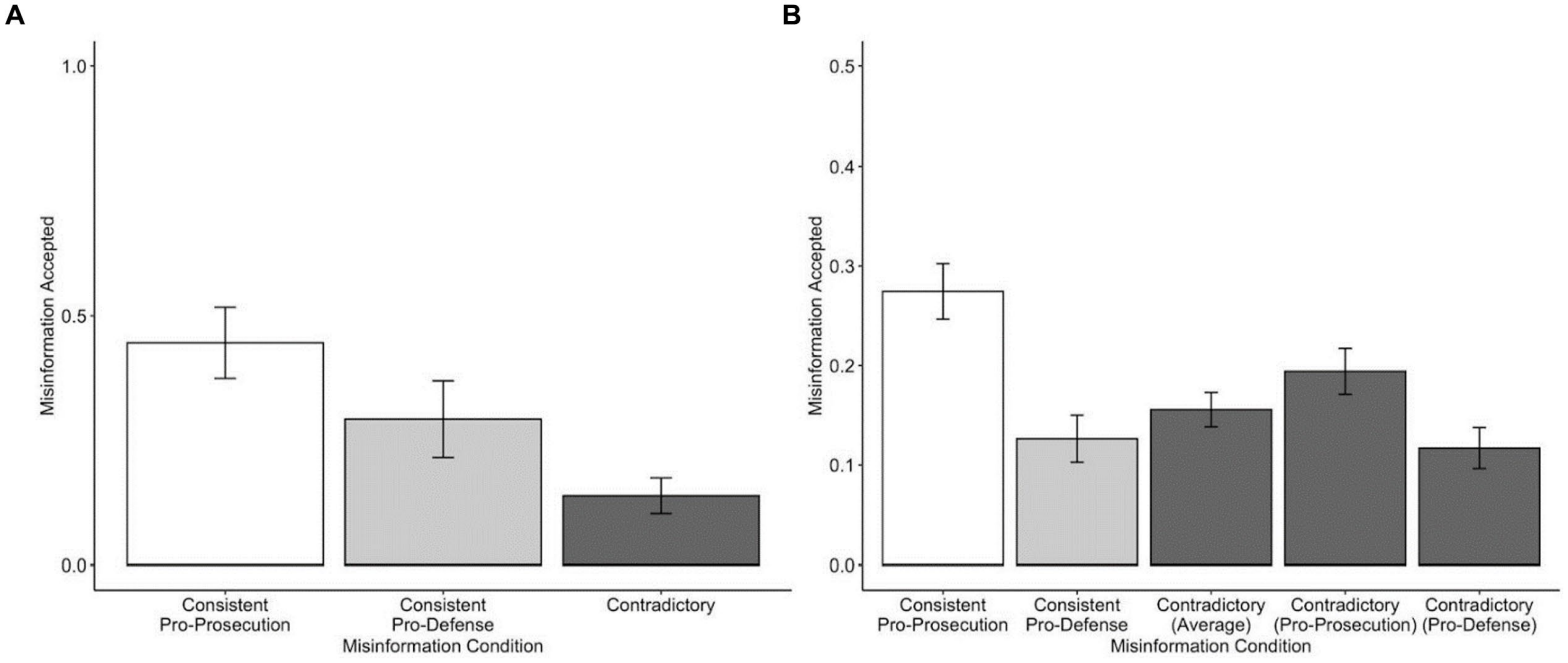
Study 1—misinformation acceptance as a function of misinformation condition in the recall **(A)** and source memory test **(B)**. **(A)** shows the total mean number of misinformation items that were misremembered as appearing during the trial during free recall across misinformation conditions. **(B)** shows the proportion of misinformation items that were misremembered as appearing during the trial during the source memory test across misinformation conditions. The “Contradictory (Average)” bar in Panel b represents the average proportion of misinformation items accepted across pro-prosecution and pro-defense items. Error bars represent standard error of the mean.

A one-way ANOVA was conducted to determine whether misinformation acceptance (i.e., reporting the misinformation from the e-deliberation) differed by misinformation condition. For misinformation acceptance, participants could receive a score ranging from 0 to 4 (as it would be implausible for participants in the contradictory condition to recall both items of misinformation for the same detail). There was a significant effect of misinformation condition on misinformation acceptance, *F*(2,272) = 5.954, *p* = 0.003, *η_p_^2^* = 0.042. As shown in Figure 1A, planned contrasts using Tukey’s HSD revealed that participants who were exposed to consistent pro-prosecution misinformation accepted more misinformation than participants who were exposed to contradictory misinformation, *t*(184) = 3.48, *p* = 0.002, *d* = 0.56, 95% CI[0.10, 0.52]. No other contrasts were significant. It should be noted that when excluding all participants who were suspicious about the deliberation (*N* = 91), misinformation condition no longer had a significant effect on misinformation acceptance.

#### 3.4.2 Source memory

Overall, 18.6% (*M* = 0.186, SD = 0.23) of misinformation items were misremembered (i.e., misinformation acceptance) as appearing during the trial. A one-way ANOVA was conducted to examine differences in misinformation acceptance between misinformation conditions. For the contradictory condition, the score entered into the ANOVA was the average misinformation acceptance score for pro-prosecution and pro-defense items. The ANOVA was significant, *F*(2,272) = 11.30, *p* < 0.001, *η_p_^2^* = 0.08. As shown in Figure 1B, planned contrasts using Tukey’s HSD procedure revealed that participants in the consistent pro-prosecution condition (*M* = 0.27, SD = 0.27) were significantly more likely to misattribute the misinformation as appearing in the trial than the consistent pro-defense group (*M* = 0.13, SD = 0.22) and contradictory group (*M* = 0.16, SD = 0.17), *t*(179) = 4.47, *p* < 0.001, *d* = 0.57, 95% CI[0.07, 0.23]; *t*(184) = 3.64, *p* < 0.001, *d* = 0.49, 95% CI[0.04, 0.19], respectively. No other contrasts were significant. However, the contrast comparing the pro-prosecution and contradictory groups was no longer significant when excluding participants who were suspicious of the deliberation (*N* = 91).

Given that there were different misinformation items (pro-prosecution and pro-defense), we conducted follow-up analyses to compare misinformation acceptance between conditions for each type of misinformation item separately. A repeated measures ANOVA revealed that participants in the contradictory condition were significantly more likely to misattribute pro-prosecution misinformation items (*M* = 0.19, SD = 0.22) as appearing in the trial than pro-defense items (*M* = 0.12, SD = 0.19), *F*(1,93) = 8.32, *p* = 0.005, *η_p_^2^* = 0.08. Furthermore, a between-subjects ANOVA revealed that participants consistently exposed to pro-prosecution items were significantly more likely to misattribute this type of misinformation as appearing in the trial than the contradictory condition (*F*[1,184] = 4.95, *p* = 0.027, *η_p_^2^* = 0.03), while participants consistently exposed to pro-defense items were no more likely to misattribute this type of information to the trial than the contradictory condition, *F*(1,181) = 0.09, *p* = 0.765, *η_p_^2^* = 0, BF₀₁ = 5.882.

## 4 Study 1 discussion

Study 1 evaluated whether different forms of misinformation introduced through jury deliberation influenced juror memory and decision-making about a common type of sexual assault case. Specifically, we compared exposure to misinformation items that consistently favored the prosecution’s case, consistently favored the defense’s case, or were contradictory (where participants received both pro-prosecution and pro-defense items). A key finding was that while exposure to misinformation during deliberation did not influence post-deliberation decision-making (e.g., verdict), it did influence participants’ memory for the trial evidence. Mock-jurors were more susceptible to accepting misinformation that aligned with the prosecution’s case than the defense’s case, particularly when multiple jurors were in agreement (i.e., consistent condition) rather than disagreement (i.e., contradictory condition) about the misinformation.

The nature of the case and the pre-deliberation decision-making of participants is likely to explain why pro-prosecution misinformation distorted memory more than pro-defense misinformation or contradictory misinformation. The current study contained an excerpt of a sexual assault trial, that only featured the complainant’s testimony of the event. We used such a case as this is reflective of many sexual assault trials, where only the complainant provides evidence-in-chief (e.g., New South Wales Law Reform Commission, 2020). The pre-deliberation decision-making measures showed a ceiling effect such that most participants (87.6%) provided a guilty verdict even before deliberation, and pre-deliberation ratings of defendant guilt and complainant credibility were high (5.5 and 5.4 out of 7, respectively). Post-deliberation verdicts and ratings were very similar to their pre-deliberation counterparts. Therefore, the greater endorsement of pro-prosecution misinformation could be explained by the fact that this misinformation most closely reflected participants’ beliefs about the case prior to deliberation, thus participants were more likely to misremember this type of information as occurring in the trial. Indeed, jury research has revealed that jurors may engage in *predecisional distortion*, where their evaluation of later case information is unconsciously influenced by the verdict that is leading in their mind (Carlson and Russo, 2001; Hope et al., 2004). The fact that most participants were leaning towards the prosecution’s case pre-deliberation might also explain why misinformation exposure did not influence decision-making post-deliberation.

However, in addition to participants’ prior beliefs about the case, social factors at the time of deliberation – such as conformity—may partially explain why pro-prosecution misinformation was accepted more in the consistent condition than the contradictory condition (Asch, 1956; Kaplan, 1984; Waters and Hans, 2009). Perhaps when participants saw that multiple jurors agreed that the misinformation was present during the trial, they felt pressure to conform with the group position. In contrast, when the jurors disagreed, participants may have felt less pressure to conform and therefore were more likely to reject the misinformation (Asch, 1956). Together, Study 1 findings suggest that cognitive, and possibly social, factors may influence misinformation acceptance during juror deliberations. More research is required, however, before solid conclusions can be made.

## 5 Study 2

Since participants’ evaluation of the trial information in Study 1 was skewed towards the prosecution’s case prior to the deliberation phase, it is difficult to determine to what extent, if any, the misinformation effect found in the pro-prosecution condition was influenced by the misinformation presented during the deliberation phase. To correct for this potential ceiling effect, in Study 2 we re-examined the effect of pro-prosecution and pro-defense misinformation on juror memory and decision-making, but with a more ambiguous sexual assault case (i.e., approximate even split of guilty and not-guilty pre-deliberation verdicts). The contradictory misinformation condition was not included in Study 2.

Another factor that might have impacted the validity of our findings in Study 1 was the high level of suspicion participants reported about the e-deliberation procedure. While our e-deliberation method did allow our participants to actively discuss the case with other ‘jurors’, just over a third of participants were suspicious about deliberation, citing that they believed it was fully simulated, that confederates were used, or that other participants were provided with alternate versions of the transcript which resulted in them receiving different information. For some analyses, results differed when the sample included versus excluded suspicious participants (e.g., free recall). Therefore, in Study 2 we used a methodology less likely to arouse suspicion in participants. Like Thorley et al. (2020), participants in Study 2 read a transcript of a deliberation, which contained misinformation about the trial evidence.

Finally, given that Study 1 revealed that the misinformation jurors are exposed to during deliberation can alter their memory for the trial, a secondary aim of Study 2 was to explore techniques to inoculate jurors from accepting misinformation mentioned during deliberation. Judicial instructions to the jury are one such technique. In a criminal trial context, jurors can be provided with instructions from the judge at the conclusion of a trial, but prior to the deliberation phase, to assist them in their decision-making. These instructions can include a range of topics, such as instructions to disregard inadmissible evidence (Steblay et al., 2006), instructions to help the jury understand legal concepts such as beyond reasonable doubt (Trimboli, 2008), and Henderson instructions to help them evaluate eyewitness testimony (Dillon et al., 2017), to name a few. Despite popular support for judicial instructions, there is mixed empirical support for their effectiveness (e.g., Alvarez et al., 2016). However, most relevant to our study, eyewitness memory studies have found that participants who received a warning about having potentially encountered incorrect post-event information about an event showed a reduced misinformation effect compared to those that received no such warning (e.g., Echterhoff et al., 2005; Blank and Launay, 2014; Bulevich et al., 2022). While most of these studies have involved post-warnings where participants received the warning after misinformation exposure, a recent study found that providing a pre-warning (warning before misinformation exposure) was also effective in reducing the misinformation effect (Karanian et al., 2020). Based on this research, we expected that participants who received a judicial instruction about the harmful effects of misinformation would be less likely to accept misinformation mentioned during deliberation than those that received no instruction. From here onwards, we use the term “judicial instruction” to refer to this warning, as this is the language used to describe such warnings given by judges in jury research.

## 6 Study 2 method

### 6.1 Participants

Four-hundred and twenty-three participants initially took part in the study. The same eligibility requirements as Study 1 were applied (over 18 years of age, Australian citizen, fluent in English). The data from 84 participants were excluded for the following reasons: failing more than one attention check (*n* = 3), not completing the study (*n* = 54), invalid data entry (*n* = 1), or spending insufficient time reading the trial transcript (as indicated by reading times that were one standard deviation below the mean reading time [*M* = 641.04 s, SD = 397.72 s], *n* = 26). After applying exclusions, 339 participants were retained in the final analyses. An *a priori* power calculation using G*Power 3.1 (Faul et al., 2007) revealed that 265 participants were needed to detect a small to medium effect (*f* = 0.20) with 90% power for a 2 × 2 between-subjects design.

Participants had a Mean age of 29.40 years (SD = 11.72) and were predominantly female (66.1%, Male = 31.3%, Non-binary/Genderqueer/Gender fluid = 2.1%, Prefer not to say = 0.6%). Most participants were of European descent (77.9%), followed by Asian (12.7%), mixed ethnicity (3.8%), and Aboriginal or Torres Strait Islander (2.4%) (Other = 3.3%). Most participants (96.2%) had never served on a jury before.

Participants consisted of undergraduate psychology students (*n* = 168) and members of the community recruited via Prolific (*n* = 171). See Table 5 for a breakdown of demographic characteristics based on recruitment strategy. Prolific participants were significantly older than psychology students, *F*(1, 337) = 145.399, *p* < 0.001, *η_p_^2^* = 0.301. There were also significant differences in participant gender between Prolific participants and psychology students, *χ^2^* (*N* = 339) = 45.856, *p* < 0.001, *φ_c_* = 0.368, with Prolific participants having a more even split between male and female participants than psychology students. There were also differences in cultural background between the samples, *χ^2^* (*N* = 339) = 47.237, *p* < 0.001, *φ_c_* = 0.373. While these demographic differences emerged, there were no differences in the frequency of psychology students and Prolific participants across the warning and misinformation conditions (both *p*s > 0.485). Additionally, there were no differences in gender or age distribution among the conditions (all *p*s > 0.250). Therefore, we will not conduct any further analyses between the two participant samples.

**Table 5:** Demographic characteristics of Study 2 participants based on recruitment strategy.

Demographics	Student participants (*n* = 168)	Prolific participants (*n* = 171)
Mean age	22.92 (6.78)	35.77 (12.07)
Gender (%)		
Female	83.3	49.1
Male	14.3	48.0
Non-binary/Genderqueer/Gender fluid	1.8	2.3
Prefer not to say	0.6	0.6
Previous jury experience (%)		
Yes	0.6	7.0
No	99.4	93.0
Cultural background (%)		
European/White	86.3	69.6
East Asian	0.6	9.9
Southeast Asian	0.6	11.1
Other	12.5	9.4

“Other” for cultural background includes cultural backgrounds with low cell counts: Aboriginal and Torres Strait Islander, African, Hispanic, Middle Eastern, Mixed, Pacific Islander, South Asian, Other. Standard deviations for mean age in parentheses.

### 6.2 Design

The current study employed a 2 × 2 between-subjects design, looking at the effects of a judicial instruction about misinformation (instruction vs. no instruction) and misinformation type (pro-prosecution vs. pro-defense) on juror memory and decision-making. Thus, participants were randomly assigned to one of four conditions: instruction/pro-prosecution (*n* = 78), instruction/pro-defense (*n* = 92), no instruction/pro-prosecution (*n* = 83), and no instruction/pro-defense (*n* = 86).

### 6.3 Materials

#### 6.3.1 Trial transcript

Similar to Study 1, participants read a shortened trial transcript depicting an alcohol-involved sexual assault. We modified the case from Study 1 to create greater ambiguity, with the goal of achieving a more even split in pre-deliberation verdicts. The transcript in Study 2 featured an opening statement from the judge and both legal parties, with the issue of consent being disputed between the parties. The alleged victim, Daphne Livingstone, was then questioned by both the prosecution and defense. Daphne’s testimony detailed that she attended her work Christmas party on the day of the alleged assault. After the Christmas party, she went to a bar with her colleague, Katie, who invited the accused, Alexander Smith, to join them. Daphne and Alexander knew each other, but had only met once before in passing. A member of their group was removed from the premises by security, and so the group went back to Katie’s house. Katie and the others in their group left to get food and drinks, leaving Daphne and Alexander alone. Daphne and Alexander kissed, and Daphne stated that she consented to this. When Alexander tried to take things further, Daphne verbally expressed that she did not want to go further as the others would be back soon. According to Daphne’s testimony, Alexander ignored this and penetrated Daphne with his penis. Daphne was shocked and did not know what to do, as she had planned to stay at Katie’s overnight and had no way of getting home. One month after the alleged assault, Daphne reported the alleged assault to police and was asked to undertake a medical examination.

At the conclusion of Daphne’s testimony, the judge then gave a closing statement. The closing statement reminded jurors of their responsibilities and the burden of proof, and provided instructions about what the jurors should consider when reaching their decision. Pertinently, we manipulated whether the judge provided a specific instruction about the possibility of encountering misinformation from other jurors during the deliberation. Specifically, participants in the instruction condition were given the following information embedded in the judge’s instructions:

“You must be reminded that during your deliberations, it is possible that other jurors will remember the facts of the case differently to you, through no fault of their own. You should be aware of the possibility that your memory of the trial may be tainted or distorted by what other jurors say during the deliberation. You should try to correct these errors during your deliberations as much as possible, so that the decision that you collectively reach is derived from the correct version of events.”

The no instruction condition did not receive the judicial instruction.

#### 6.3.2 Deliberation transcript

Many participants were suspicious that the deliberation was fake in Study 1, and this suspicion had to be considered when interpreting the results. To mitigate suspicion in Study 2, we presented participants with a transcript of a fictional deliberation between four jurors, similar to Thorley et al. (2020). Participants were either provided with pro-prosecution or pro-defense misinformation for four of the details in the deliberation. Each juror in the transcript provided one misinformation item and one correct item from the trial during the deliberation. As in Study 1, we selected central misinformation items that, if remembered, would be likely to impact decisions on the case. The misinformation items targeted were those that related to common misconceptions and stereotypes about sexual assault, including the relationship between the complainant and defendant, the actions of the complainant during the assault, the time to report the assault, and the presence of physical injuries (see Carr et al., 2014; Australian Institute of Health and Welfare (AIHW), 2020). Two of the misinformation items were contradictory (i.e., contradicted the correct information from the trial) and two were additive (i.e., referred to details that were not mentioned in the trial). Table 6 presents misinformation and correct information provided by the simulated jurors based on experimental condition, and correct details for questions where misinformation was provided.

**Table 6:** Misinformation and correct items in Study 2 based on misinformation condition.

Item	Pro-prosecution	Pro-defense	Correct
Misinformation			
Status of the relationship	Strangers—had never met before	Friends—had met several times before	Acquaintances—had met once before*
Behavior during assault	Attempted to push Alex off	Did not push Alex off	N/A*
Reporting of assault	One day after	Two months later	One month later*
Presence of injuries	Bruises consistent with being held down	No bruises consistent with being held down	N/A*
Correct			
Doing the day of assault			Attending a work Christmas party
Why they left the bar			Friend removed from premises for spilling a drink
Why the complainant stayed			She had no way of getting home
Information requested by police			Undertake a medical examination

^*^Indicates correct details from the trial that were not featured in any version of the deliberation. These details are provided in the table to compare to the misinformation items. N/A refers to additive misinformation items (i.e., there was no reference to these details in the trial transcript).

Participants were asked two questions about their perceptions of the deliberation. First, they were asked to rate the extent they believed the deliberation would be similar to the discussions that real jury members would have in a real deliberation of a sexual assault case (from 1 to 7, where 1 = *not at all similar* and 7 = *extremely similar*). Second, they were asked to rate how accurate they believed the jurors in the deliberation were in their memory of the information from the trial. An error in the formatting of the response options for this question emerged, therefore responses to the accuracy question will not be considered in the analyses.

### 6.4 Measures

#### 6.4.1 Verdict and guilt ratings

Like Study 1, participants were asked to render a verdict of *guilty* or *not guilty* for the defendant with a justification, and rate the defendant’s guilt, both pre- and post-deliberation. Participants also rated the perceived strength of the prosecution and defense cases at pre- and post-deliberation (on a scale from 1 to 7, where 1 = *weak*, 4 = *uncertain*, and 7 = *strong*). Pre- and post-deliberation decision-making occurred before and after misinformation exposure, respectively.

#### 6.4.2 Credibility

Like Study 1, participants were asked to rate their perception of the complainant’s honesty, believability, credibility, and accuracy both pre- and post-deliberation. There was high internal consistency in pre- and post-deliberation ratings for all four items (pre-deliberation: *Cronbach’s α* = 0.946; Post-deliberation: *Cronbach’s α* = 0.961). Therefore, like in Study 1, these ratings were aggregated to form a single pre-deliberation and post-deliberation complainant credibility score.

#### 6.4.3 Recall memory

Participants completed both a free recall and a cued recall memory questionnaire. For the free recall memory task, participants were given an open-ended prompt and asked to recall what they remember about the alleged sexual assault case described in the trial transcript. They were specifically instructed to only report facts about the case (i.e., what the complainant alleged happened), as opposed to any of the instructions they were provided by the judge. They were also instructed to base their responses off their own memory of the events, and encouraged to report everything they could remember while being as accurate and detailed as possible. Participants were given unlimited time to complete free recall.

The cued recall questionnaire was added to Study 2, to more precisely measure misinformation acceptance. The questionnaire consisted of 12 specific questions about the case, presented to participants in a randomized order. The cued recall questions were selected so that four questions focused on case facts where misinformation was provided during the deliberation (the relationship, time to report, events during alleged assault, information provided to police), four questions focused on case facts where correct information was provided during the deliberation (events of the day, why they left the bar, what the complainant did after alleged assault, information sought by police), and four questions focused on case facts that were not mentioned during the deliberation (discussion at the bar, why the parties were left alone, information being disputed, what the parties did at the bar). Participants were encouraged to rely only on their own memory of the trial when answering the cued recall questions, and to be as accurate and detailed as possible.

##### 6.4.3.1 Recall coding

The same coding system from Study 1 was employed, whereby independent scorers coded participant responses using the categories of misinformation accepted, misinformation rejected, and correct accepted. Using these categories, Scorer 1 (HC) coded 100% of participant responses in both free and cued recall. Scorer 2 (SB) coded 51% of free recall responses, and Scorer 3 (GR) coded 51% of cued recall responses. For both free and cued recall, the Intraclass Correlation Coefficients revealed good to excellent reliability for all coding categories (all *ICC*s > 0.836). Given the acceptable reliability, the coding from Scorer 1 was used in the analyses.

#### 6.4.4 Source memory

Like Study 1, Study 2 included a source memory test. Participants were given the same instructions as they were given in Study 1. The source memory test in Study 2 consisted of 36 items. Eight of the items related to the misinformation from the deliberation; the correct answer to these items depended on the misinformation condition participants were assigned, like Study 1 (*Deliberation only* if a misinformation item was relevant to their experimental condition, or *Neither* if a misinformation item was not relevant to their experimental condition). There were 4 items related to the correct information from the deliberation (correct answer: *Both trial and deliberation*), 8 items related to correct information not covered in the deliberation (correct answer: *Trial only*), and 16 new filler items (correct answer = *Neither trial nor deliberation*). The total number of critical source memory errors (i.e., misinformation acceptance) was the key dependent variable which was calculated in the same way as the consistent conditions in Study 1 (i.e., “Trial Only” and ‘Trial and Deliberation’ scores for the misinformation items were summed together). Since participants were exposed to the same number of misinformation items in Study 2, we did not calculate proportion scores. Analyses for whether there were differences across misinformation and instruction condition with regard to correct (trial only), correct (trial and deliberation), and new source memory items (16 filler statements +4 statements relating to misinformation that they were not exposed to) are provided in the Supplementary Data Sheet S1.

#### 6.4.5 Rape myth acceptance

As the misinformation items in Study 2 reflected common misconceptions about sexual assault that may most likely be introduced during deliberations in such cases, it is possible that participants with greater rape myth acceptance may be most susceptible to reporting misinformation. Therefore, in Study 2, participants completed the adapted version of the Illinois Rape Myth Acceptance Scale—Subtle Version (IRMA-S; Thelan and Meadows, 2022) to assess rape myth acceptance. We included the 22-items from the IRMA-S that assessed rape myth acceptance, but did not include the filler items. Participants indicated their agreement with each statement on a 5-point Likert scale (where 1 = “*strongly disagree*” and 5 = “*strongly agree*”). Scores on each item were summed to form a total score of rape myth acceptance. Reverse scoring was applied for three of the items. Possible scores ranged from 22 to 110, with higher scores indicating greater rape myth acceptance. The IRMA-S has high internal consistency (*α* = 0.93) and good validity when evaluated with diverse participant samples. The adapted version we used in the current study also had high internal consistency (*α* = 0.88).

#### 6.4.6 Attention and manipulation checks

Like Study 1, we included several attention and manipulation checks. There were three instructional manipulation checks spread throughout the study; participants were required to answer at least two of these questions correctly for their data to be retained in the data analysis. Additionally, we were interested in determining whether participants who received the judicial instruction about being exposed to misinformation during the deliberation remembered receiving this instruction. Memory for the judicial instruction was measured in two ways. First, participants were asked to summarize the judicial instruction in their own words. We coded participants’ responses based on whether they mentioned being warned about potential for misinformation to occur in the deliberation or not. Second, participants were asked three yes/no questions about whether the judge had provided a warning about three different topics. Two of these questions related to distractor topics (unreliability of physical evidence and burden of proof), whereas the other question asked whether participants were warned about memory being tainted by other jurors. Collectively, these manipulation checks provided useful information on the effectiveness of the judicial instruction and assessed whether participants understood the judicial instruction. Participants were also asked what they believed the purpose of the study was to probe suspicion about the aims of the study.

### 6.5 Procedure

Participants took part in Study 2 online. Given the sensitive nature of the case, participants were provided the contact details of support services before being asked to read the trial transcript. Like Study 1, participants were told to read the transcript in full and not to make any notes while reading the transcript. The transcript was split into separate pages on the online survey host, and we recorded the time participants spent on each page. After reading the transcript, participants completed the pre-deliberation decision-making measures (verdict, guilt rating, strength of case ratings, complainant credibility ratings). Participants were then required to read a transcript of a fictitious deliberation about the case and to imagine that they are forming part of the jury on this case and are involved in the discussion. As with the trial transcript, participants were told to read the deliberation transcript in full and to not make any notes. The deliberation transcript contained either pro-prosecution or pro-defense misinformation, depending on the condition participants had been randomly assigned to. Participants completed the same measures of decision-making post-deliberation. Then, they completed the free recall, cued recall, and source memory tasks. Next, participants answered two questions about their perceptions of the deliberation. They then completed the adapted version of the IRMA-S, following which they completed the manipulation checks. Finally, participants provided demographic information and were debriefed about the study. All aspects of Study 2 were approved by the University of Newcastle Human Research Ethics Committee (protocol number: H-2022-0079).

### 6.6 Transparency statement

We reported how we determined our sample size, all data exclusions (if any), all manipulations, and all measures in this study. The hypotheses, design, measures, and analysis plan were pre-registered on OSF. The registration can be found at https://osf.io/vcwkj/. Below we note deviations from our pre-registration transparently. All experimental materials and data (dataset, output, and code) are available on the OSF: https://osf.io/wdse5/.

### 6.7 Transparent deviations

First, after commencing data collection for this study we realized that the survey software we used to host this study was not randomizing participants to all experimental conditions. This meant we had data in some cells and not in others. We recruited more participants to even up the number of participants in each condition (to ensure we could conduct our planned analyses without violating assumptions). For this reason, we exceeded our pre-registered sample size.

Second, we did not pre-register any specific data exclusion criteria focused on checking whether participants had spent sufficient time on the trial transcript pages to ensure that they had read the materials. As the experimental manipulations were contained in the trial materials, it is critical that participants read the materials properly. After data was collected, three authors who had not had contact with the data (ND, FN, GR) decided that it would be reasonable to exclude participants who had a total average reading time of more than one standard deviation below the overall sample total average reading time as participants who viewed the trial materials for this period of time were unlikely to have properly read the materials.

Third, we also planned to look at whether perceptions of the accuracy of the jurors in the deliberation predicted misinformation acceptance, but an error with the programming of the scale anchors for this question meant that the question likely did not make sense to participants. For this reason, we have not analyzed this data as planned.

## 7 Study 2 results

### 7.1 Overview and analysis plan

First, we reported on the results of the manipulation check relating to the judicial instruction manipulation using descriptive statistics (paraphrase test) and chi-square analyses (forced choice). Then, we conducted a series of mixed-methods ANOVAs to determine whether there was any effect of misinformation type and judicial instruction on changes in decision-making (from pre- to post-deliberation), as well as to ensure that there were no existing differences in decision-making prior to the deliberation. We conducted two logistic regressions (for pre- and post-deliberation verdict) to determine whether misinformation type and judicial instruction influenced verdicts. Next, we conducted several two way ANOVAs to determine whether misinformation and judicial instruction conditions influenced participants’ memory and misinformation acceptance in all three memory tasks (free recall, cued recall, source memory). We conducted moderation analyses to determine whether rape myth acceptance moderated the strength of the relationship between misinformation condition and misinformation acceptance. We also conducted mediation analyses to determine whether the relationship between misinformation condition and post-deliberation decision-making was mediated by misinformation acceptance. Finally, we conducted a regression to determine whether perceptions of the realism of the deliberation predicted misinformation acceptance (see Supplementary Data Sheet S1 for results of this analysis). Like Study 1, we reported Bayes Factors alongside the frequentist analyses.

### 7.2 Manipulation checks

To check the memorability of the judicial instruction about encountering misinformation during deliberation, participants completed a paraphrase test (summarizing the judge’s instructions in their own words) and answered a yes/no question indicating whether the judge provided such a warning. The paraphrase test revealed that only 11 participants (6.5%) in the instruction condition reported the misinformation instruction. No participants in the no instruction condition spontaneously reported the misinformation instruction. When participants were asked to state whether the judge had warned them about potentially encountering misinformation during the deliberation, 74.7% of participants in the instruction condition correctly responded “yes,” compared to 36.1% of participants in the no instruction condition incorrectly responding “yes.” The chi-square analysis revealed a significant relation between judicial instruction condition and responses to this manipulation check, with “yes” responses above expected counts for the instruction condition, and below expected counts for the no instruction condition, *χ^2^* (1, *N =* 339) = 51.148, *p* < 0.001, *φ_c_* = 0.388, BF₁₀ > 100.

### 7.3 Decision-making

At pre-deliberation, 66.7% of participants delivered a verdict of “guilty” and 33.3% of participants delivered a verdict of “not guilty.” After deliberation, 61.4% of participants delivered a verdict of “guilty” and 38.6% of participants delivered a verdict of “not guilty”.

Two hierarchical logistic regressions were conducted—one for pre-deliberation and one for post-deliberation—to determine whether misinformation and instruction conditions predicted verdicts. In block 1 of each model, we added the main effects of misinformation type and instruction, and in block 2, we added the misinformation × instruction interaction. As Table 7 demonstrates, misinformation type, instruction, and the interaction did not significantly predict pre-deliberation verdicts, but misinformation type did predict verdicts post-deliberation. Specifically, the odds of delivering a guilty verdict following the deliberation were 2.982 times higher in the pro-prosecution condition compared to the pro-defense condition. A Bayesian chi-square also revealed extreme evidence for a relationship between misinformation condition and post-deliberation guilt ratings, BF₁₀ > 100.

**Table 7:** Study 2—hierarchical logistic regressions for pre- and post-deliberation verdicts with misinformation and judicial instruction conditions as predictors.

	*B*	S.E.	Sig.	OR	95% CI
Pre-deliberation					
**Block 1**					
Misinformation	0.082	0.232	0.722	1.086	0.690, 1.710
Instruction	−0.340	0.232	0.142	0.712	0.452, 1.121
**Block 2**					
Misinformation × Instruction	−0.370	0.465	0.426	0.691	0.278, 1.717
Post-deliberation					
**Block 1**					
Misinformation	1.092	0.235	< 0.001*	2.982	1.880, 4.728
Instruction	0.193	0.231	0.403	1.213	0.771, 1.908
**Block 2**					
Misinformation × Instruction	0.296	0.471	0.529	1.345	0.535, 3.383

Verdict was coded as 0 (not guilty) and 1 (guilty). Pro-prosecution misinformation was coded as 0 and pro-defense misinformation was coded as 1. No warning was coded as 0 and warning as 1. OR = odds ratio. 95% confidence intervals are for odds ratio scale.^*^p < 0.05.

Mixed methods ANOVAs were conducted to determine whether misinformation and instruction conditions impacted decision-making (guilt ratings, complainant credibility, strength of case) both pre- and post-deliberation. Table 8 provides the descriptive statistics accompanying these analyses. For all decision-making measures, there was a significant time × misinformation interaction. Specifically, there were no differences in guilt ratings, complainant credibility ratings, and the perceived strength of the prosecution and defense cases between the pro-prosecution and pro-defense misinformation conditions before the deliberation (all *p*s > 0.312, all BF₀₁s > 5.131). After the deliberation, participants in the pro-prosecution misinformation condition gave significantly higher ratings of defendant guilt (using Tukey’s LSD: *p* = 0.016, BF₁₀ = 2.033). None of the other pairwise comparisons between pro-prosecution and pro-defense misinformation at post-deliberation were significant after applying Tukey’s LSD (all *p*s > 0.088, all BF₀₁s > 1.933). Participants in the pro-defense misinformation condition showed a significant decrease in ratings of guilt and complainant credibility from pre- to post-deliberation (*p*s < 0.001, BF₁₀s > 100), and a significant increase in ratings of the strength of the defense’s case (*p* < 0.001, BF₁₀ = 7.980), but there was no significant change in ratings of the strength of the prosecution’s case from pre- to post-deliberation (*p* = 0.087, BF₀₁ = 2.327). For participants in the pro-prosecution condition, ratings of the strength of the prosecution’s case significantly increased from pre- to post-deliberation (*p* < 0.001, BF₁₀ = 73.944), with no other significant differences emerging after applying Tukey’s LSD (all *p*s > 0.083, BF₀₁s > 1.367). Taken together, these findings suggest that exposure to different forms of misinformation may alter decision-making from pre- to post-deliberation in the direction of the legal party the misinformation favors. This appears to be particularly the case for decision-making related to guilt (verdicts and guilt ratings).

**Table 8:** Study 2—pre- and post-deliberation verdict, defendant guilt rating, complainant credibility rating, and strength of evidence ratings across misinformation conditions.

	Pre-deliberation		Post-deliberation		Pre-post deliberation		Misinformation × time interaction			
	Pro-prosecution (*N* = 161)	Pro-defense (*N* = 178)	Pro-prosecution (*N* = 161)	Pro-defense (*N* = 178)	Pro-prosecution (*N* = 161)	Pro-defense (*N* = 178)	*F*	*p*	*η^2^*	*BF₁₀*
% Guilt	65.84	67.42	74.53	49.44	–	–	–	–	–	
Defendant guilt rating	5.30 (1.64)	5.41 (1.48)	5.44 (1.58)	5.00 (1.69)	−0.13 (0.85)	0.41 (1.04)	27.20	0.001	0.007	>100
Complainant credibility	5.39 (1.24)	5.52 (1.11)	5.43 (1.22)	5.25 (1.26)	−0.04 (0.51)	0.27 (0.57)	27.46	0.001	0.004	>100
Strength of prosecution’s case	4.77 (1.71)	4.86 (1.64)	5.05 (1.72)	4.74 (1.59)	−0.27 (0.93)	0.12 (0.99)	14.20	0.001	0.004	15.140
Strength of defense’s case	2.90 (1.53)	2.84 (1.52)	2.82 (1.52)	3.10 (1.52)	0.08 (0.77)	−0.26 (1.15)	10.25	0.002	0.003	3.188

Values for defendant guilt rating, complainant credibility, strength of prosecution’s case, and strength of defense’s case represent mean rating (standard deviation in parentheses).

### 7.4 Memory

For misinformation acceptance in free and cued recall, there was no significant effect of misinformation type (free recall: *F*[1,335] = 0.196, *p* = 0.658, *η^2^ < 0*.001, BF₀₁ = 11.212; cued recall: *F*[1,335] = 0.196, *p* = 0.658, *η^2^ < 0*.001, BF₀₁ = 9.872), instruction (free recall: *F*[1,335] = 0.157, *p* = 0.692, *η^2^ < 0*.001, BF₀₁ = 11.535; cued recall: *F*[1,335] = 2.461, *p* = 0.118, *η^2^ = 0*.007, BF₀₁ = 4.008), and no significant misinformation type × instruction interaction (free recall: *F*[1,335] = 0.230, *p* = 0.632, *η^2^ < 0*.001, BF₀₁ = 101.248; cued recall: *F*[1,335] = 2.614, *p* = 0.107, *η^2^* = *0*.008, BF₀₁ = 15.589). However, for critical source memory errors, there was a significant main effect of misinformation type (*F*[1,335] = 4.513, *p* = 0.034, *η^2^* = *0*.013), with participants in the pro-defense condition (*M* = 1.39, SD = 0.95) misattributing the misinformation to the trial more than participants in the pro-prosecution condition (*M* = 1.17, SD = 1.07). However, the Bayes Factor indicated ambiguous evidence for a lack of difference, BF₀₁ = 1.467. There was no significant effect of instruction (*F*[1,335] = 3.857, *p* = 0.050, *η^2^* = *0*.011, BF₀₁ = 1.936) and no misinformation type × instruction interaction (*F*[1,335] = 0.016, *p* = 0.898, *ηp^2^ < 0*.001, BF₀₁ = 6.543) for critical source memory errors. Mediation analyses conducted in JASP also revealed that misinformation acceptance in any of the memory tasks did not mediate the relationship between misinformation type (pro-prosecution vs. pro-defense) and any of the post-deliberation decision-making outcomes (all *p*s > 0.362).

We also explored whether source misattributions were more likely to occur when exposed to the misinformation (as opposed to spontaneous misattributions of misinformation from the other condition). This was the case. These analyses are reported in the Supplementary Data Sheet S1.

### 7.5 Rape myth acceptance

The average score on the adapted version the IRMA-S was 40.85 (SD = 11.80), with a minimum score of 22 and a maximum score of 84 (higher scores indicating greater rape myth acceptance). We used a series of linear regression analyses to determine whether rape myth acceptance was a significant moderator in the relationship between misinformation type and misinformation acceptance. As rape myth acceptance was continuous, scores were mean centered using a z-transformation. For both free and cued-recall, rape myth acceptance was not a significant moderator (both *p*s > 0.856). However, for source memory, rape myth acceptance was a significant moderator of the effect of misinformation type on critical source memory errors, *β* = −0.172, *t*(3,335) = −2.213, *p* = 0.028. Simple effects analyses were conducted by looking at the relationship between rape myth acceptance scores and critical source memory errors for pro-prosecution and pro-defense participants separately. Rape myth acceptance significantly predicted critical source memory errors in the pro-prosecution condition, *β* = 0.233, *t*(1,159) = 3.015, *p* = 0.003, but did not predict critical source memory errors in the pro-defense condition, *β* = 0.008, *t*(1,176) = 0.107, *p* = 0.915.

## 8 General discussion

Across two studies, we evaluated whether different forms of misinformation introduced during jury deliberation influenced mock-juror memory and decision-making in sexual assault trials. In addition, Study 2 explored whether being warned about the harmful effects of misinformation during judicial instructions would inoculate mock-jurors from accepting misinformation presented during deliberation. In general, Study 1 revealed that pro-prosecution misinformation was more likely to be accepted as appearing in the trial than pro-defense misinformation, particularly when jurors were in agreement (i.e., consistent condition) rather than in disagreement (i.e., contradictory condition). Misinformation type did not influence decision-making. Using a more ambiguous sexual assault trial transcript to address the unequal split of pre-deliberation verdicts in Study 1, Study 2 found a limited effect of misinformation on memory, where participants in the pro-defense condition were more likely to misattribute the misinformation to the trial than participants in the pro-prosecution condition (source memory only). The relationship between misinformation condition and critical source memory errors was also moderated by rape myth acceptance. In contrast to Study 1, Study 2 did find an effect of misinformation type on decision-making. That is, after deliberation, being exposed to pro-defense misinformation led to a decrease in ratings of defendant guilt, complainant credibility, and an increase in the strength of the defense’s case. However, there was no effect of the judicial instruction about misinformation on participants’ decision-making.

### 8.1 The effect of misinformation on juror memory

Decades of research into the misinformation effect in an eyewitness context has found that exposure to misinformation can distort later memory (see Loftus, 2005; Frenda et al., 2011, for reviews). Building on this literature, our findings showed that jurors too may be vulnerable to the misinformation effect; the findings from both studies revealed that if jurors encounter misinformation in jury deliberations, they may make source monitoring errors and come to misattribute the misinformation as evidence presented during the trial (Johnson et al., 1993). Our findings align with Thorley et al. (2020), who also found that when participants were exposed to misinformation through a written deliberation, they misattributed the misinformation as trial evidence, compared to participants who were not exposed to misinformation.

The current research expanded on Thorley et al.’s (2020) study by exploring the effects of both pro-prosecution and pro-defense misinformation on juror memory. Our findings revealed that the type of misinformation that our mock-jurors misremembered as trial evidence varied across studies. Participants in Study 1 were more likely to misremember pro-prosecution misinformation as forming part of the trial than pro-defense misinformation, while the opposite was true for Study 2. The differences in findings between studies may be partly attributable to differences in methodology. As we noted above, the greater endorsement of pro-prosecution over pro-defense misinformation in Study 1 could be because participants’ beliefs about the case were skewed towards the prosecution’s case prior to deliberation. In Study 2, we developed a case vignette with a more even split of pre-deliberation verdicts, finding that participants were more likely to misremember pro-defense than pro-prosecution misinformation as trial evidence (source memory only). Participants’ tendency to accept pro-defense misinformation over pro-prosecution misinformation might be explained by laypeople’s general mistrust of rape allegations (Webster et al., 2018; Minter et al., 2021). The pro-defense misinformation items were designed to discredit the prosecution case. Thus, perhaps participants more readily endorsed the pro-defense misinformation as evidence from the trial because these items aligned with people’s attitudes towards rape allegations, whereas the pro-prosecution items did not.

In Study 2, we found that higher endorsement of rape myths was positively associated with source memory errors for participants exposed to pro-prosecution misinformation. This could be explained by the fact that the pro-prosecution misinformation items in Study 2 aligned with several pervasive rape myths. Take, for example, the following items from the IRMA-S (Thelan and Meadows, 2022) used in our study: *“If a woman does not physically fight back, she cannot really say she was raped”* and *“Sexual assault probably did not happen if the woman has no bruises or marks.”* In Study 2, participants were not presented with any information in the trial about whether the victim fought back or had bruises/marks. However, the pro-prosecution misinformation items for these facts were worded in the same direction as the rape myths (*“Attempted to push off”*/“*Bruises consistent with being held down*”), whereas the pro-defense misinformation items were worded in the opposite direction to the rape myths (“*Did not push off”*/*“No bruises consistent with being held down”*). Therefore, it is logical that participants who endorsed these rape myths (i.e., had higher rape myth acceptance scores) were more likely to misattribute the misinformation as having appeared in the trial transcript. This suggests that rape myths, which some scholars argue function as a schema for how sexual violence occurs (Süssenbach et al., 2012), are influencing participants’ memories and views of the case. Problematically, most sexual assaults do not occur in ways that are consistent with rape myths (Dinos et al., 2015).

Other research on the misinformation effect shows similar findings. For instance, research that has explored the phenomenon of fake news has shown that people are more likely to misremember fake news when it aligns with their existing beliefs on the topic (e.g., Abortion: Murphy et al., 2019; Feminism: Murphy et al., 2021)—a finding referred to as *ideological congruency.* This idea of accepting misinformation that is more in line with one’s pre-existing beliefs, similar to confirmation bias, may also explain why pro-prosecution misinformation was more likely to be accepted in Study 1; most participants already believed in the defendant’s guilt before exposure to misinformation, and the pro-prosecution misinformation (serving to give credibility to the complainant’s case) may have strengthened these beliefs.

The fact that we only found misinformation effects for source memory and not recall memory in Study 2 might be explained by the type of memory evoked by the different memory tests. The source memory test simply required participants to recognize and then endorse/not endorse statements. When participants were presented with the pro-defense misinformation statements, it might have activated their attitudes about rape, leading them to ‘recognize’ these details as forming a part of the trial evidence. The recall tests required participants to actively retrieve information about the trial. The cues provided by these questions might not have been enough to elicit information pertaining to the misinformation. Additionally, recall and recognition-based memory tasks place different demands on the individual’s ability to monitor and control the information provided (Koriat and Goldsmith, 1996). Specifically, individuals are often more accurate in recall compared to recognition memory tasks, often at the expense of providing fewer details. Confidence plays an important role in how individuals respond, as recall memory tasks allow individuals to withhold low-confidence responses, while recognition memory tasks do not. Measuring memory confidence in future studies will allow us to determine whether differences in misinformation acceptance across tasks can be explained through strategic regulation processes. Notwithstanding, considering misinformation acceptance using both recall and recognition-based memory tasks is a strength of our study, as previous research studies looking at misinformation in jury contexts have mostly only employed source memory tasks (e.g., Ruva and McEvoy, 2008; Ruva and Guenther, 2015; Thorley et al., 2020), and looking at only source memory does not give a full picture of how misinformation exposure in discussion settings leads to memory conformity.

### 8.2 The effect of misinformation on juror decision-making

In Thorley et al. (2020), participants were presented with pro-prosecution misinformation during the deliberation phase. They found that the more pro-prosecution misinformation participants attributed to the trial, the more likely they were to give a guilty verdict. We were interested in determining whether misinformation that favors the defense would have the opposite effect, through increased acquittals and unfavorable perceptions of the complainant’s case. While we found no evidence of misinformation effects on decision-making in Study 1 (likely due to ceiling effects in decision-making at pre-deliberation), Study 2 revealed a pattern consistent with our hypotheses. From pre- to post-deliberation, participants’ decision-making tendencies (e.g., guilty vs. not guilty) shifted based on the type of misinformation they were exposed to during deliberation. Specifically, misinformation that discredited (i.e., pro-defense misinformation) rather than strengthened the prosecution’s case had a more pronounced effect on decision-making—exposure to pro-defense misinformation led to less favorable perceptions of the prosecution’s case (e.g., decreases in conviction rates, ratings of defendant guilt, complainant credibility).

We expected that misinformation acceptance would be the mechanism by which exposure to different types of misinformation would affect legal decisions. In other words, we expected that the effect of the type of misinformation on post-deliberation decision-making would be mediated by participants’ distorted memory about the trial evidence. We did not find any evidence that misinformation acceptance mediated this relationship. Interestingly, this means that the mechanism by which exposure to incorrect trial facts during the deliberation affects post-deliberation decision-making is not one of memory. This is in contrast to previous research looking at the effects of pre-trial publicity on mock-juror memory and decision-making, where critical source memory errors mediated the relationship between exposure to pre-trial publicity and guilt ratings (Ruva et al., 2007; Ruva and Guenther, 2015). However, an alternative mediator that we did not explore in the current study was evidence interpretation (Ruva and Guenther, 2015), such that exposure to different forms of pre-trial publicity appears to alter the way in which trial facts are interpreted (i.e., which legal party they favor), which in turn influences decision-making. It would be useful in future research to also consider the role of evidence interpretation in the relationship between misinformation exposure during deliberation and post-deliberation decision-making.

### 8.3 Judicial instructions

Although there is popular support for judicial instructions to reduce jurors’ reliance on extra-legal factors, there is mixed evidence for their effectiveness (e.g., Alvarez et al., 2016). In Study 2, we found that the judicial instruction had no effect on mock jurors’ memory errors or decisions made about the trial. This is consistent with research which indicates that many judicial instructions do not reduce jurors’ reliance on extra-legal factors like curative instructions for pre-trial publicity (e.g., Steblay et al., 2006) or Henderson instructions about evaluating eyewitness testimony (e.g., Dillon et al., 2017).

There are several barriers which can prevent judicial instructions from assisting jurors in their decision-making. First, if jurors do not understand what they are being told to do in the instruction, then they were not able to apply the instruction to their decision-making (Baguley et al., 2020). In Study 2, we checked (mock) jurors’ comprehension of the judicial instructions about misinformation using a paraphrase test in which participants were asked to summarize the judge’s instructions in their own words. We found that only 6.5% of participants mentioned the instruction about misinformation in their responses. When we explicitly asked participants whether they remembered receiving an instruction from the judge about misinformation, 25% of participants said they did not remember this. Collectively, these results suggest that the judge’s instruction about the effect of misinformation, while understood, was perhaps not salient. This may explain why the instruction did not affect either memory outcomes or decision-making in Study 2.

A second barrier to judicial instructions helping jurors in their decision-making is if the strategy offered to assist the jury in the instruction is ineffective (Baguley et al., 2020). The instruction we used adopted two common strategies used in instructions that target bias — making the jury aware of the potential bias and encouraging them to challenge inaccurate information they heard from other jurors. Given participants could not actively challenge misinformation from other jurors as the deliberation in Study 2 was presented as a transcript, perhaps the instruction may be effective when participants engage in an interactive or live deliberation. That said, in other research on jury deliberations, jurors find it difficult to contradict information presented by other jurors when they think it is wrong (e.g., Stasser and Titus, 2003). An important avenue for future research would be to test the effectiveness of this instruction when participants engage in a more interactive deliberation.

### 8.4 Limitations and future directions

There are a few limitations of the current research to consider. The methods we used to simulate jury deliberation in our studies lacked ecological validity. While our e-deliberation method in Study 1 did allow our participants to actively discuss the case with other ‘jurors’, most participants were suspicious about deliberation. In Study 2, we adopted the same methodology as Thorley et al. (2020) where participants read a transcript of a deliberation. While this method allowed us to account for suspicion, it lacks ecological validity as participants did not actively participate in the deliberation. Despite this, many participants in Study 2 still reported the transcript of the deliberation to be similar to the type of discussions they believed real jurors would have (see Supplementary Data Sheet S1). The fact that we found that the type of misinformation did influence mock juror memory even in our less interactive deliberations suggests that it will be important to examine misinformation effects in more interactive jury deliberations. For example, future research might consider holding discussions in-person or via video conferencing where a confederate introduces misinformation about the trial evidence. The use of confederates to implant misinformation in a consistent fashion has been used extensively in the eyewitness memory research around co-witness discussion (e.g., Gabbert et al., 2004; Paterson et al., 2009; Eisen et al., 2017), and could be effective in creating a realistic jury deliberation that arouses little suspicion. Alternatively, participants could be exposed to two different versions of the trial transcript and naturally introduce misinformation. Such an approach has also been used successfully in the eyewitness memory literature (e.g., Gabbert et al., 2003, 2006, 2007; Paterson et al., 2011).

There are several important avenues for future research arising from this work. One is investigating how participant characteristics, in particular participant gender, might affect how (mock) jurors remember case evidence and make decisions in sexual assault cases. While many individual studies provide evidence of participant gender effects on decisions made in sexual assault cases, a recent review suggests that evidence for participant gender effects is mixed and context dependent (Gravelin et al., 2019). Many investigations of participant gender in decision-making in sexual assault cases are exploratory (e.g., Nitschke et al., 2021) which may mean that studies are underpowered to adequately detect the interaction of participant gender and other factors in the study (e.g., Giner-Sorolla, 2018). An important avenue for future research in this area will be to investigate potential participant gender effects on acceptance of misinformation in jury deliberations in sexual assault cases.

Another question for future research concerns the efficacy of a warning from the judge to prevent misinformation during jury deliberations from affecting memory and decision-making. In Study 2, we found that the warning delivered via judicial instruction did not seem to assist jurors to avoid misinformation during deliberations. Research suggests that judicial instructions can be revised to make them more effective (e.g., Steblay et al., 2006). Future research should consider whether the content of the judicial instruction can be made more effective by drawing on the research literature to support accurate memory. As with warnings to eyewitnesses, the timing of a judicial instruction can also be important to whether it assists the jury (e.g., Alvarez et al., 2016). Future research should investigate when and how many times the judicial instruction needs to be given to help jurors avoid misinformation during deliberation.

Finally, both of our studies explored the impact of different types of misinformation on mock-juror memory and decision-making in a sexual assault trial. We chose sexual assault as the offense because of the diverse attitudes that jurors may hold and thus bring into deliberations, making it particularly important to understand the consequences of misinformation exposure in these cases. Future research should determine whether our findings generalize to other crime types, as well as whether misinformation introduced about other forms of evidence can similarly influence memory and decision-making (e.g., forensic experts, eyewitnesses).

## 9 Conclusion

Across two studies, our findings provide initial insight into the effect of misinformation exposure during jury deliberations on juror memory. The findings suggest that jurors may misremember trial details when exposed to misinformation provided by fellow jurors, but that this memory distortion may depend on the nature of the misinformation items, the consistent repetition of these misinformation items, and the beliefs and attitudes held about legal cases and how certain offences occur (e.g., rape myths). Future research should continue to consider the important role of memory in jury deliberation contexts and the factors that increase or decrease memory distortion due to misinformation exposure. With a clearer understanding of memory conformity effects in criminal trial settings, we can determine to what extent memory distortion subsequently biases legal decision-making.

## Data availability statement

The datasets presented in this study can be found in online repositories. The names of the repository/repositories and accession number(s) can be found below: Study 1: https://osf.io/wqgsm/, Study 2: https://osf.io/wdse5/.

## Ethics statement

The studies involving humans were approved by the University of Sydney Human Research Ethics Committee (protocol number: 2019/947) and the University of Newcastle Human Research Ethics Committee (protocol number: H-2022-0079). The studies were conducted in accordance with the local legislation and institutional requirements. The participants provided their written informed consent to participate in this study.

## Author contributions

HC, ND, FN, and GR contributed to funding acquisition, data curation, methodology, project administration, and validation for the current project, contributed to the conceptualization and investigation of Study 1, contributed to the formal analysis of Study 1, and contributed to the original manuscript preparation and writing. HC, ND, FN, GR, and NW contributed to the conceptualization and investigation of Study 2. HC, GR, and SB contributed to the formal analysis of Study 2. ND contributed to data visualization in Study 1. HC contributed to data visualization in Study 2 and developed software and was involved in supervision for the current project. HC and ND provided resources for the current project. All authors contributed to the article and approved the submitted version.

## Footnotes

^1^ https://osf.io/wqgsm/
