# Supplementary material for: The impact of misinformation presented during jury deliberation on juror memory and decision-making
Source: Front Psychol. 2024 Jan 26;15:1232228. doi: 10.3389/fpsyg.2024.1232228 (PMC10853387; doi:10.3389/fpsyg.2024.1232228)
Supplement: Supplementary file 1 [file Data_Sheet_1.docx]

**Supplemental file: The impact of misinformation presented during jury deliberation on juror memory and decision-making**

**Study 1**

***Source memory***

Three between-subjects ANOVAs were conducted to compare the proportion of correct information (both trial and deliberation), correct (trial only), and new items that were remembered/misremembered as appearing during the trial across the three misinformation conditions (see Table S1 for descriptive statistics). Overall, 82% (*M* = .82, *SD* = .19) of correct information (both trial and deliberation) items, 76.6% (*M* = .76, *SD* = .27) of correct (trial only) items, and 15% (*M* = .15, *SD* = .13) of new items were remembered/misremembered as appearing during the trial. The three ANOVAs analysing the different items were not significant, suggesting that all three misinformation conditions equally remembered the correct information (both trial and deliberation) (*F*[2,272] = .48, *p* = .62, *η_p_^2^* = .003), correct (trial only) (*F*[2,272] = .09, *p* = .91, *η_p_^2^* = .001), and new (*F*[2,272] = .74, *p* = .48, *η_p_^2^* = .005) information as appearing during the trial. Additionally, the Bayes factor for each information type indicated that there was strong evidence in favor of the null hypothesis (correct [both trial and deliberation]: BF_10_ = 0.06; correct [trial only]: BF_10_ = 0.04; new: BF_10_ = 0.08).

**Table S1**

*Study 1 - The Proportion of Correct Information (Both Trial and Deliberation), Correct (Trial Only) Information, and New Information that Participants (Mis)remembered as Appearing During the Trial As a Function of Misinformation Condition*

|  | Misinformation Condition, *M*(*SD*) | | |
| --- | --- | --- | --- |
| Information Type* | Pro-Prosecution (n = 92) | Pro-Defense  (n = 89) | Contradictory (Average) (n = 94) |
| Correct (Both Trial and Deliberation) | .83 (.18) | .82 (.19) | .80 (.19) |
| Correct (Trial Only) | .76 (.26) | .78 (.29) | .76 (.26) |
| New | .14 (.11) | .16 (.14) | .14 (.13) |

*Note.* The correct (both trial and deliberation) and correct (trial only) proportion scores were computed based on 4 items each. The new proportion score was computed based on 8 items.

***Perceived Accuracy of Jurors***

The first 29 participants who completed the study provided two separate ratings: one for each of the fellow jurors in the deliberation. The remaining participants completed just a singular rating for the perceived accuracy of their fellow jurors. To provide an equivalent rating for both sets of participants, an average rating for the former participants was compiled. It should be noted that out of these 29 participants, only four provided different ratings of perceived accuracy for the two jurors. These four participants were in the contradictory condition. Despite this, given the low frequency of participants in the contradictory condition who reported differing accuracy ratings for the two jurors (4%), we believed that compiling these two questions to form an aggregate score would be unlikely to meaningfully impact upon the pattern of results.

A one-way ANOVA was conducted to determine whether the perceived accuracy of the other deliberating jurors (combined) differed based on misinformation condition. The ANOVA was significant, *F*(2,260) = 8.572, *p* < .001, *η_p_^2^* = .062. Contrasts using Tukey’s HSD revealed that the perceived accuracy of the other jurors in the pro-prosecution condition (*M* = 4.17, *SD* = 1.21) was significantly greater than the perceived accuracy of the other jurors in the pro-defense condition (*M* = 3.69, *SD* = 1.16), *p* = .019), and contradictory condition (*M* = 3.46, *SD* = 1.12), *p* < .001. However, there was no difference in the perceived accuracy of the other jurors in the pro-defense and contradictory conditions, *p* = .402. It should also be noted that when excluding participants who were suspicious about the deliberation (*N* = 91), the contrast comparing pro-prosecution and pro-defense conditions on perceived credibility of the jurors was no longer significant.

Two linear regressions were conducted to determine whether the perceived accuracy of the other deliberating jurors predicted the number/proportion of misinformation items accepted in the free recall and source memory test, respectively. For the source memory data, the average misinformation acceptance score was used in the analysis. The regression analyzing free recall data revealed that perceived accuracy of the jurors did not predict acceptance of misinformation, *R^2^* = .013, *F*(1,261) = 3.453, *p* = .064, BF₀₁ = 1.446. The regression analyzing the source memory data revealed that perceived accuracy of the jurors significantly predicted acceptance of misinformation, *R^2^* = .136, *F*(1,261) = 41.18, *p* < .001. We further examined whether the association between juror accuracy and misinformation acceptance differed based on misinformation condition, however there was no evidence of this, *p*s > .67. Overall, this suggests that the more accurate participants’ viewed jurors during deliberation, the more likely they were to accept the misinformation items in the source memory test.

**Study 2**

***Source memory***

Three between-subjects ANOVAs were conducted to compare the proportion of correct information (both trial and deliberation), correct (trial only), and new items that were remembered/misremembered as appearing during the trial across the misinformation and judicial instruction conditions (see Table S2 for descriptive statistics). Overall, 87% (*M* = .87, *SD* = .18) of correct information (both trial and deliberation) items, 79% (*M* = .79, *SD* = .16) of correct (trial only) items, and 13% (*M* = .13, *SD* = .11) of new items were remembered/misremembered as appearing during the trial. For correct (both trial and deliberation) and new items, there were no significant main effects or interactions (all *p*s > .28, all BF₀₁ > 8.420). For correct (trial only) items, there was no significant main effects of misinformation or instruction (*p*s > .481, BF₀₁ > 7.085), but there was a significant interaction between misinformation and instruction conditions, *F*(1,335) = 5.459, *p* = .020, *η_p_^2^* = .016. However, the Bayes Factor indicated moderate evidence in favor of no interaction, BF₀₁ = 6.523. Simple effects analyses revealed that for the pro-prosecution condition, there was no significant effect of instruction on correct attributions of trial only items, *F*(1,159) = 1.450, *p* = .230, *η_p_^2^* = .009. However, for participants in the pro-defense condition, the effect of instruction on correct attributions of trial only items was significant, *F*(1,176) = 4.372, *p* = .038, *η_p_^2^* = .024. As Table S2 indicates, for participants in the pro-defense condition, those who received the judicial instruction about misinformation were *less* likely to correctly attribute trial only statements to the trial than participants who did not receive the instruction.

**Table S2**

*Study 2 - The Proportion of Correct Information (Both Trial and Deliberation), Correct (Trial Only) Information, and New Information that Participants (Mis)remembered as Appearing During the Trial As a Function of Misinformation and Instruction Condition*

|  |  | Misinformation Condition, *M*(*SD*) | | |
| --- | --- | --- | --- | --- |
| Information Type* | Pro-Prosecution  (n = 161) | | Pro-Defense  (n = 178) | |
|  | No Instruction  (n = 83) | Instruction  (n = 78) | No Instruction  (n = 86) | Instruction  (n = 92) |
| Correct (Both Trial and Deliberation) | .85 (.20) | .89 (.16) | .88 (.16) | .88 (.18) |
| Correct (Trial Only) | .78 (.15) | .81 (.14) | .81 (.15) | .76 (.18) |
| New | .14 (.10) | .12 (.09) | .12 (.12) | .12 (.12) |

*Note.* The correct (both trial and deliberation), correct (trial only), and new proportion scores were computed based on 4, 8, and 20 items, respectively.

As participants were given statements that related to the misinformation they encountered as well as misinformation for the opposing condition that they were never actually exposed to, we also explored whether source misattributions were more likely to occur when exposed to the misinformation (as opposed to spontaneous misattributions of misinformation from the other condition). In essence, the statements relating to misinformation that the participant was not exposed to acted as control statements. Analyses revealed that participants in the pro-prosecution condition attributed more pro-prosecution misinformation to the trial than participants in the pro-defense condition (*F*[1,337] = 29.338, *p* < .001, *η²* = .080), and participants in the pro-defense condition attributed more pro-defense misinformation to the trial than participants in the pro-prosecution condition (*F*[1,337] = 29.120, *p* < .001, *η²* = .080). Therefore, source misattributions were greater when exposed to the misinformation than spontaneous misattributions.

***Realism of Deliberation***

Participants were asked how similar they believed the deliberation transcript would be to the actual discussions jurors would partake in during real deliberations. On average, participants believed the deliberation transcript was moderately similar to a real jury discussion (*M* = 4.30, *SD* = 1.50). Linear regression analyses revealed that perceived realism of the deliberation did not predict misinformation acceptance in any of the memory tasks (all *p*s > .162, all BF₀₁ > 3.257).
